# United We Fall: All-or-None Forgetting of Complex Episodic Events

**DOI:** 10.1037/xge0000648

**Published:** 2019-07-15

**Authors:** Bárður H. Joensen, M. Gareth Gaskell, Aidan J. Horner

**Affiliations:** 1 Department of Psychology, University of York; 2 Department of Psychology, and York Biomedical Research Institute, University of York

**Keywords:** episodic memory, forgetting, statistical modeling, hippocampus

## Abstract

Do complex event representations fragment over time, or are they instead forgotten in an all-or-none manner? For example, if we met a friend in a café and they gave us a present, do we forget the constituent elements of this event (location, person, and object) independently, or would the whole event be forgotten? Research suggests that item-based memories are forgotten in a fragmented manner. However, we do not know how more complex episodic, event-based memories are forgotten. We assessed both retrieval accuracy and dependency—the statistical association between the retrieval successes of different elements from the same event—for complex events. Across 4 experiments, we show that retrieval dependency is found both immediately after learning and following a 12-hr and 1-week delay. Further, the amount of retrieval dependency after a delay is greater than that predicted by a model of independent forgetting. This dependency was only seen for coherent “closed-loops,” where all pairwise associations between locations, people, and objects were encoded. When “open-loops” were learned, where only 2 out of the 3 possible associations were encoded, no dependency was seen immediately after learning or after a delay. Finally, we also provide evidence for higher retention rates for closed-loops than for open-loops. Therefore, closed-loops do not fragment as a function of forgetting and are retained for longer than are open-loops. Our findings suggest that coherent episodic events are not only retrieved, but also forgotten, in an all-or-none manner.

How are complex episodic events forgotten? Research on forgetting has primarily focused on the rate at which it occurs (Ebbinghaus, 1913; Rubin & Wenzel, 1996), and whether forgetting occurs via interference or decay (McGeoch, 1932). However, the question of how memory traces change as a function of forgetting has received less attention. For example, are memory traces forgotten in an all-or-none, holistic manner, or do they instead fragment over time, such that some aspects of the memory trace are forgotten more quickly than others?

Early research on forgetting was dominated by a theoretical debate concerning whether forgetting occurs as a function of interference; where overlapping memory traces disrupt one another, or decay; where memory traces decay over time (see Wixted, 2004 for a review of the forgetting literature). Evidence of greater forgetting of nonsense syllables when participants remained awake, relative to when participants slept, between study and test were taken as evidence for interference accounts (Jenkins & Dallenbach, 1924), as decay was thought to lead to equal rates of forgetting across wake and sleep. As more interfering material would be encoded in the awake, relative to sleep, condition following learning, the greater rate of forgetting for participants who remained awake was taken as evidence for retroactive interference. Further evidence for the interference account was provided by Underwood (1957). However, Underwood showed that the more material learned prior to the critical test information, the greater the subsequent forgetting. Thus, contrary to the Jenkins and Dallenbach (1924) findings, forgetting appeared to occur predominantly as a function of proactive interference (see Postman, 1971 for a review of the interference literature).

More recently, the idea that forgetting is principally a function of interference, and proactive interference in particular, has been questioned. For example, reviewing much of the traditional psychological literature, Wixted (2004) proposed that forgetting is predominantly a result of retroactive interference from mental activity or new memory formation more generally, as opposed to the specific interference that occurs from learning related/overlapping material. Further, although initially rejected (McGeoch, 1932), the concept of memory decay has also been revived (Frankland, Köhler, & Josselyn, 2013; Hardt, Nader, & Nadel, 2013). This account holds that some forgetting will be due to the deterioration of the biological substrates of the memory trace itself. Importantly, both proposals draw on our neuroscientific understanding of forgetting and the concept of consolidation; where new memory traces are thought to stabilize over time, becoming less susceptible to interference and/or decay (see Dudai, Karni, & Born, 2015 for a review of the consolidation literature).

Despite this resurgent interest in forgetting, relatively little research has focused on whether mnemonic representations change as a function of forgetting. Although, dual-process memory models have proposed different rates of forgetting dependent on the type of representations (Brainerd & Reyna, 2002; Reyna & Brainerd, 1995) or that different representations are more likely to be forgotten via decay or interference (Sadeh, Ozubko, Winocur, & Moscovitch, 2014), here we ask whether mnemonic representations change as a function of forgetting. Specifically, do mnemonic representations fragment over time, or are they instead more likely to be forgotten in an all-or-none manner?

Brady, Konkle, Alvarez, and Oliva (2013) recently used forgetting rates to infer the representational structure of item-based memory traces. They found different forgetting rates for separate aspects of an object (i.e., color and state). Specifically, the color of an object was forgotten more rapidly than the state of the object (i.e., its pose or configuration of parts). The results suggest that item-based memories fragment over time, with some aspects of the memory trace being forgotten more rapidly than others. They also assessed retrieval dependency—the statistical relationship between retrieval successes for the two aspects associated with the same object. The presence of dependency has previously been used to infer the coherence of the underlying mnemonic representation (Horner & Burgess, 2013, 2014). Consistent with previous research (Meiser & Bröder, 2002; Starns & Hicks, 2008), Brady et al. (2013) saw evidence for dependency after initial encoding, but importantly dependency decreased over time. This decrease in dependency is consistent with a fragmentation of the memory trace as a function of forgetting.

Here, we asked whether more complex event-based memories also fragment over time. Whereas, item-based memories can be supported by medial temporal lobe regions outside of the hippocampus, such as the perirhinal cortex (Aggleton & Brown, 1999; Diana, Yonelinas, & Ranganath, 2007), the hippocampus is critical to the encoding and retrieval of event-based memories (Cohen & Eichenbaum, 1993; O’Keefe & Nadel, 1978; Scoville & Milner, 1957; Squire & Zola-Morgan, 1991; Vargha-Khadem et al., 1997). Thus, these two types of mnemonic representations rely on distinct regions of the medial temporal lobe (though see Song, Wixted, Hopkins, & Squire, 2011; Wais, Wixted, Hopkins, & Squire, 2006). This point is critical given the recent proposal that forgetting processes may differ between these two regions (Sadeh et al., 2014). Indeed, recent research has suggested that whereas event-based memories/recollection, supported by the hippocampus, are more likely to be forgotten via decay processes; item-based memories/familiarity, supported by the perirhinal cortex, are more likely to be forgotten via interference (Sadeh, Ozubko, Winocur, & Moscovitch, 2016). Given these dissociations, it is possible that event-based memories do not undergo the same fragmentation process seen in Brady et al. (2013) but are instead forgotten in a more all-or-none manner. In contrast to recent evidence showing simultaneous reductions in overall accuracy and dependency as an effect of negative valence items at encoding (Bisby, Horner, Bush, & Burgess, 2018), a lack of a decrease in dependency, despite reductions in accuracy, would be consistent with evidence showing retained dependency for source features associated with words despite decreases in overall accuracy as a function of dual task load (Boywitt & Meiser, 2013).

We have previously shown that the encoding of overlapping pairwise associations can result in retrieval dependency (Horner & Burgess, 2014). For example, if a participant learns associations between *kitchen* and *hammer*, *kitchen* and *Barack Obama*, and *hammer* and *Barack Obama* across three separate encoding trials, retrieval dependency is seen for all constituent elements of this separately encoded ‘event.’ If you are cued with *kitchen* and successfully retrieve *Barack Obama*, you are more likely to also successfully retrieve *hammer* when cued with *kitchen* on a separate retrieval trial. This dependency is similar to that seen when all three elements are encoded on a single trial (Horner & Burgess, 2013, 2014), suggesting that encoding all three pairwise associations forms a coherent event engram similar in nature to that formed in a single spatiotemporal context. We have also provided fMRI evidence that these elements are bound into coherent event engrams in the hippocampus, allowing for the subsequent retrieval of all event elements (Horner, Bisby, Bush, Lin, & Burgess, 2015). The retrieval of all event elements is consistent with the idea that recollection (as opposed to familiarity) is associated with all-or-none, or holistic, retrieval (Yonelinas, 1994), and provides evidence for all-or-none retrieval occurring via pattern completion; the complete retrieval of a representation (i.e., pattern) in the presence of a partial or ambiguous cue (Gardner-Medwin, 1976; Hopfield, 1982; Marr, 1971; McClelland, McNaughton, & O’Reilly, 1995; Treves & Rolls, 1992; for reviews, see Horner & Doeller, 2017; Hunsaker & Kesner, 2013). Consistent with this, Meiser, Sattler, & Weisser (2008) showed that dependency for source details (i.e., location and size), associated with words, is observed when participants report subjective experiences of recollection, but not when reporting feelings of familiarity.

Here, we define *forgetting* as decreases in accuracy between retrieval time points, and remain theoretically agnostic as to whether forgetting is a result of decreased accessibility for intact memory traces, or a loss of the underlying trace itself (Tulving & Pearlstone, 1966). Although any measure of forgetting will inevitably be derived from differences in the proportion of memories retained between two retrieval time points, here we ask, as these coherent events are forgotten (as measure by retrieval accuracy), do we see decreases in retrieval dependency? This would imply that the underlying memory traces are fragmenting over time (see Figure 1C). However, if forgetting occurs, but dependency is consistent over time, then this would imply that coherent event-based memories are instead forgotten in an all-or-none manner (see Figure 1B), with event-based memories being more likely to be either retained or forgotten in their entirety.[Fig-anchor fig1]

We used a design similar to that developed by (Horner & Burgess, 2014). Across all four experiments, at encoding, participants learned a series of multielement events (see Figure 2). Each event consisted of three elements (locations, famous people and objects). Events were ‘built up’ over two/three separate, spaced, encoding trials. Each trial consisted of the presentation of one of the three possible pairwise associations from an event. This allows us to build events with different structures of overlapping pairs: closed-loops, where all the pairwise associations are encoded (e.g., *kitchen*–*hammer*, *kitchen*–*Barack Obama*, *hammer*–*Barack Obama*) or open-loops, where only two out of the three possible pairwise associations are encoded (e.g., *kitchen*–*hammer*, *kitchen*–*Barack Obama*).[Fig-anchor fig2]

We have previously shown that dependency is seen for closed-loops (and three element events learned on a single encoding trial), but not for open-loops (Horner et al., 2015; Horner & Burgess, 2013, 2014). The associative structure formed for closed-loops is therefore similar in nature to a coherent event engram formed in a single encoding trial. Given these findings, we refer to *closed-loop associative structures* as “events” but note that they are not single spatiotemporal events as typically defined. Dependency is not seen for open-loops and, as such, the open-loop condition serves as a control condition where dependency is not expected, even when retrieval shortly follows encoding. The inclusion of the open-loop condition also allowed us to assess the further possibility that overlapping associations may undergo a process of integration over time, such that open-loops might show dependency after a delay. This is in light of research showing that the ability to infer the relationship between nonencoded B–C pairs (after encoding A–B and A–C pairs) increases following a short nap (Lau, Tucker, & Fishbein, 2010). Thus, sleep may play a role in generalizing across related, but independently encoded, information (Ellenbogen, Hu, Payne, Titone, & Walker, 2007; Wagner, Gais, Haider, Verleger, & Born, 2004), as is the case for open-loops.

At immediate and delayed retrieval, we tested the encoded associations from half of the events in both directions (e.g., cue location, retrieve the associated person; cue person, retrieve the associated location) using cued six-alternative forced choice. In Experiment 1, we tested immediately and following a delay of 12 hr. Forgetting was presumed to have occurred after 12 hr relative to the immediate condition. Given the well-established finding that sleep decreases forgetting (see Diekelmann & Born, 2010 for a review), we also manipulated the extent of forgetting by training in the morning or evening, such that half the participants were awake between study and test and half were asleep. The sleep manipulation also allowed us to assess whether sleep played a role in integrating pairwise associations encoded as open-loops (e.g., Lau et al., 2010).

Experiment 1 showed clear evidence for retained retrieval dependency in the closed-loop condition after a 12-hr delay, even in the awake condition where forgetting was high. Given the dependency seen for closed-loops, we further assessed dependency following a week (in Experiment 2 through 4). Across all four experiments, we see variable rates of forgetting, but no evidence for a change in dependency for closed-loops (or open-loops). A lack of a decrease in dependency for closed-loops, despite a decrease in overall memory performance, shows that closed-loops retain their dependency after forgetting has occurred, implying that forgetting is more likely to occur in an all-on-none manner with closed-loops being either retained or forgotten in their entirety.

## Experiment 1

Experiment 1 assessed retrieval accuracy and dependency immediately after encoding and after a 12-hr delay. We manipulated the time of encoding, such that half the participants encoded pairwise associations in the morning and half encoded in the evening. This meant that half the participants slept between study and test (sleep condition), and half were awake between study and test (awake condition). The inclusion of a sleep manipulation was twofold: (1) to vary the amount of forgetting while controlling for the interval between initial learning and subsequent testing and (2) to assess whether sleep plays a role in the integration of two overlapping pairwise associations (i.e., open-loops).

Lau et al. (2010) found that when participants learned overlapping A–B and A–C pairs (i.e., open-loops), their ability to infer a relationship between B and C increased following a nap, relative to an awake condition. However, B–C inference can potentially occur via two means: (1) encoding generalization, where prior to retrieval, A–B and A–C associations are integrated into a generalized representation that potentially forms a direct association between B and C (Shohamy & Wagner, 2008; Zeithamova, Dominick, & Preston, 2012) or (2) retrieval generalization, where the relationship between B and C is inferred “on-the-fly” at the point of retrieval (Banino, Koster, Hassabis, & Kumaran, 2016; Kumaran & McClelland, 2012). Assessing retrieval accuracy and dependency for open-loops allows us to differentiate between these two possibilities, under the assumption that if A–B and A–C pairs are integrated prior to retrieval, behavioral dependency will be seen. Thus, if we see increases in B–C inference as a function of sleep, with an associated increase in dependency, this would support encoding (or nonretrieval) based generalization. If B–C inference increases without any increase in dependency, this would support retrieval-based generalization. In the latter case, sleep might increase the associative strength of the directly encoded A–B and A–C pairs, and this might subsequently increase the probability of B–C inference at retrieval.

Open-loops served as a control condition for closed-loops in relation to assessing forgetting of coherent (closed-loop) event-based memories. However, given evidence for the possible integration of open-loops as a result of sleep (Lau et al., 2010), we also focused on potential increases in dependency in the open-loop condition. In short, Experiment 1 asked (1) whether dependency for closed-loops decreases over time (in relation to our core question of whether coherent events fragment as a function of forgetting) and (2) whether dependency for open-loops increases over time (in relation to whether overlapping associations are integrated as a function of sleep).

### Method

#### Participants

From previous published work (Horner et al., 2015; Horner & Burgess, 2013, 2014), with *N* = 177, we calculated an effect size of *d* = .62 on our ability to detect a significant difference between the proportion of joint retrieval in the data and independent model (see the following Modeling retrieval dependency section). Using G*Power (Faul, Erdfelder, Buchner, & Lang, 2009), we conducted a power analysis with *d* = .62 and α= .05 and computed that we required a sample size of 26 to detect a significant effect, if one is present, with a power of .85.

One hundred four participants (26 participants per condition, across four between-subjects conditions) gave informed consent to participate in Experiment 1. Participants were recruited from the University of York student population and took part in exchange for course credit or monetary compensation. Participants took part in one of four conditions: 26 participants in the open-loop, awake condition (23 female, *M* age = 19.88 years, age range = 18–28 years), 26 in the open-loop, sleep condition (19 female, *M* age = 19.68, age range = 18–23 years), 26 in the closed-loop, awake condition (23 female, *M* age = 20.15 years, age range = 18–25), and 26 in the closed-loop, sleep condition (20 female, *M* age = 20.65, age range = 18–28 years). All studies were approved by the Department of Psychology Ethics Committee, University of York.

#### Materials

The stimuli consisted of 60 locations (e.g., *kitchen*), 60 famous people (e.g., *Barack Obama*), and 60 common objects (e.g., *hammer*; available at http://osf.io/k495x/). From these, 60 randomized location-person-object triplets were generated for each participant. Note, we use *triplet* to refer to the three elements (location, person and object) that were assigned to the same associative structure (closed- or open-loop). Triplets were randomly assigned across the experimental conditions open- versus closed-loops. For closed-loops, all three possible pairwise associations for a given triplet were encoded. For open-loops only two out of the three pairwise associations were encoded. Triplets were never presented all together at study or test. Only specific pairwise associations were encoded and retrieved for each triplet, dependent on whether they were open- or closed-loops. Triplets were randomly assigned to the within-subject experimental conditions tested (i.e., tested at Time 1 [T1]) versus not-tested (i.e., not tested at T1; results are reported in the online supplemental material).

Note, the open-loop condition is equated to the closed-loop condition in the number of elements, but not in terms of the number of associations. We have previously shown that a lack of dependency for open-loops is seen when three overlapping associations are encoded in an associative chain (e.g., *kitchen*–*hammer*, *kitchen*–*Barack Obama*, *Barack Obama*–*dog*), controlling for the number of associations (but not the number of elements) between open- and closed-loops (Horner & Burgess, 2014). Any differences in dependency between the two conditions in the current experiments are therefore unlikely to be driven by differences in the number of associations. Although we control for the exposure to each pairwise association across open- and closed-loops, two of the individual elements in the open-loop condition are only presented once, whereas all elements are presented twice in the closed-loop condition. Controlling for exposure to each element would require repetition of pairwise associations in the open-loop condition. We prefer to control for the number of exposures to each pairwise association, given this is what is being tested at retrieval, rather than the number of exposures to each individual element. The open- and closed-loop structures are similar in nature to the structures encoded to induce the fan effect, where RTs increase and accuracy decreases in a cued recall task as the number of elements (e.g., locations) associated with one element (e.g., person) is increased (Anderson, 1974). However, here we used event-unique locations, people, and objects for both the closed- and open-loop structures, minimizing the likelihood of inducing a fan effect.

#### Procedure

The experiment consisted of a single encoding session and two test sessions. Self-report ratings of alertness were collected before encoding and the second test session using the Stanford Sleepiness Scale (results reported in the online supplemental material). Session 1 (T1) took place between approximately 8 a.m. and 9 a.m. for participants in the awake condition (open-loop: *M* = 8:31 a.m., range = 7:57–9:19 a.m.; closed-loop: *M* = 8:46 a.m., range = 8:04–9:31 a.m.) and approximately 8–9 p.m. for participants in the sleep condition (open-loop: *M* = 8:52 p.m., range = 8:05–9:34 p.m.; closed-loop: *M* = 8:48 p.m., range = 7:48–9:34 p.m.). T1 consisted of a single study phase, and a test phase (see details to follow). Participants in the awake conditions spent the remainder of the day normally and returned approximately 12 hr later for session 2 (Time 2 [T2]; open-loop: *M* = 11 hr, 50 min, range = 11 hr, 40 min–12 hr, 7 min; closed-loop: *M* = 11 hr, 49 min, range = 11 hr, 28 min–12 hr, 3 min) at approximately 8 to 9 p.m. Participants in the post-encoding sleep condition returned to their own residence, slept overnight, and returned approximately 12 hr later (open-loop: *M* = 11 hr, 51 min, range = 11 hr, 23 min–12 hr, 16 min; closed-loop: *M* = 11 hr, 53 min, range = 11 hr, 44 min–12 hr, 13 min) at approximately 8 to 9 a.m. A 2 × 2 (Loop × Sleep) between-subjects analysis of variance (ANOVA), with the factors Loop referring to whether participants encoded open- or closed-loops and Sleep referring to whether encoding was followed by sleep or wakefulness, revealed no significant difference in the duration of the interval between T1 and T2 across conditions (*F*s < 2.00, *p*s > .16).

Participants in the sleep condition completed a sleep diary prior to T1 and T2. Self-reported sleep durations were not collected from three participants in the open-loop condition and one participant in the closed-loop condition. Sleep quality ratings were not collected from three participants in the open- and closed-loop condition, respectively. We found no differences in self-reported duration, *t*(46) = .22, *p* = .83, *d* = .06, or quality, *t*(44) = .54, *p* = .59, *d* = .16, of sleep between T1 and T2 for participants in the open versus closed-loop sleep conditions.

##### Encoding (T1)

During encoding, participants were presented with specific pairwise associations for each of the 60 triplets. Participants learned one pairwise association per trial. All pairwise associations were presented on a computer screen as words, with one item to the left and one to the right of fixation. The left/right assignment was randomly chosen on each trial. The words remained on screen for 6 s. Participants were instructed to imagine, as vividly as possible, the items interacting in a meaningful way for the full 6 s. For example, when presented with the words *Barack Obama* and *hammer*, they might imagine Obama accidently hitting his thumb with a hammer. Each word-pair presentation was preceded by a 500-ms fixation cross and followed by a 500-ms blank screen. In the open-loop condition, participants learned, for each triplet, two (out of the three possible) pairwise associations, making a total of 120 encoding trials. For each triplet in the closed-loop condition, participants learned all three pairwise associations, making a total of 180 encoding trials.

The encoding phase consisted of two or three blocks, for the open- and closed-loops respectively, of 60 trials with one pair from each triplet being presented during each block (participants were not made aware of this structure). A break of 20 s would follow every 30 encoding trials. Within each block, the order of presentation was randomized. Each open-loop consisted of a common item (e.g., if the participants learned location-person and then location-object, location would be the common item). Twenty triplets were randomly assigned to each of the three possible common items (i.e., locations, people or objects). The presentation order for open-loops across the two blocks was (1) person-location, location-object; (2) location-object, object-person; (3) object-person, person-location. Closed-loops were randomly rotated in the same manner. The presentation order for the closed-loops across the three encoding blocks was: (1) person-location, location-object, object-person; (2) location-object, object-person, person-location; (3) object-person, person-location, location-object.

##### Test (T1 and T2)

During the test sessions, participants performed a forced-choice cued-recognition task. On a given trial, the cue and six possible targets were presented simultaneously on screen. The cue was presented in the middle of the screen with six possible targets; one target and five foils from the same category (e.g., if the target word was hammer, the five foils would be other randomly selected objects from other triplets), in two rows of three below the cue. Participants had 6 s to respond with a key press and were instructed to be as accurate as possible in the time given. The location of the correct target item was randomly selected on each retrieval trial. Missing responses (*M* = .05, *SD* = .07) were counted as incorrect trials for both the accuracy and dependency analyses. A 2 × 2 (Loop × Sleep) between-subjects ANOVA, where the dependent variable was the proportion of nonresponses (collapsed across T1 and T2), showed no significant effects (*F*s < 2.5, *p*s > .11). Thus, any differences in dependency across conditions are unlikely to be caused by assuming nonresponses would have been incorrect. Note also that due to the 6-alternative forced choice recognition test, the chance of guessing correctly was relatively low (∼16.7%).

For T1, 30 out of 60 triplets were tested. Each triplet was tested with one of the cue–target associations (e.g., cue: person, target: location) in both directions. For the open-loop condition, cue–target associations were presented across four blocks (with a single, randomly assigned, pairwise association from each triplet tested in each block), making a total of 120 trials. Only the directly encoded pairwise associations for open-loops were tested at T1 (i.e., no inference test was performed). For the closed-loop condition, the associations were presented across six blocks (i.e., three pairwise associations, tested in both directions, randomly assigned across blocks), making a total of 180 trials. A 20 s break would follow every 30 trials. At T2, participants performed the same cued-recognition task as during T1 with all the triplets tested, making a total of 240 and 360 trials for the open- and closed-loop condition, respectively.

For the open-loop condition, participants performed an additional inference test following the main cued-recognition task at T2. For example, if a participant had encoded the pairwise associations between *Barack Obama* and *hammer* and *hammer* and *kitchen*, the nonencoded association between *Barack Obama* and *kitchen* would be tested in both directions (i.e., cue: *Barack Obama*, retrieve: *kitchen*; cue: *kitchen*, retrieve: *Barack Obama*) during the inference task. For the inference task, the nonencoded associations for each open-loop were tested, in each direction, across two blocks, making a total of 120 trials. A 20 s break would follow every 30 trials. Participants were not explicitly told that these were inference trials and carried out the task in the same manner as for directly encoded pairs.

In the main analysis comparing T1 versus T2, we only used retrieval trials at T2 for triplets that were not tested at T1 in order to control for possible testing effects. We include further analyses that directly compare retrieval accuracy and dependency at T2 for previously tested triplets versus triplets tested for the first time (reported in the online supplemental material).

#### Modeling retrieval dependency

Six independent 2 × 2 contingency tables for the observed data and independent model were created for each participant in order to assess the dependency between the retrieval of two items (e.g., person, object) when cued by a common item (e.g., location) A_B_A_C_, and between the retrieval of a common item (e.g., location) when cued by the other two items (e.g., person, object) B_A_B_A_. Once constructed, we calculated the proportion of joint retrieval and joint nonretrieval in the data and independent model for each contingency table separately, by summing the leading diagonal cells and dividing by the total number of events (i.e., the proportion of events where two overlapping pairwise associations within an event were both retrieved either correctly or incorrectly). We then averaged this measure across the six contingency tables to provide us with a single measure of the proportion of joint retrieval and nonretrieval for the data and independent model separately. For brevity, we refer to this measure as the “proportion of joint retrieval,” but note that it includes both the proportion of joint retrieval and joint nonretrieval.

The independent model assumes that pairwise associations for a given event are retrieved independently of one another—that is, if you retrieve one pairwise association from an event (un)successfully this does not predict your ability to (un)successfully retrieve another pairwise association from the same event. As such, the independent model serves as a lower bound which we can compare with the proportion of joint retrieval in the data. Note, that the proportion of joint retrieval measure scales with accuracy, and as such only comparisons between the data and independent model (i.e., the “dependency” measure reported in the following text) are meaningful.

The 2 × 2 contingency tables for the data shows the number of events that fall within the four cells (i.e., for the A_B_A_C_ analysis, both A_B_ and A_C_ correct; A_B_ incorrect and A_C_ correct; A_B_ correct and A_C_ incorrect; and both A_B_ and A_C_ incorrect, where A_B_ = cue with location (A) and retrieve person (B) and similarly for A_C_, where C stands for object). The table for the independent model (see Table 1) shows the predicted proportion of events that fall in the four cells, given a participant’s overall level of accuracy, if the retrieval of within-event associations is assumed to be independent. For a given participant, the proportion of correct retrievals of, for instance, item B when cued by A is denoted by *P*_AB_ (i.e., the mean performance for B when cued by A across all events). For the independent model, when cued by A, the probability of (1) correctly retrieving both B and C (across all events) is equal to *P*_AB_*P*_AC_; (2) correctly retrieving B but not C is equal to *P*_AB_ (1 − *P*_AC_); (3) correctly retrieving C but not B is equal to (1 – *P*_AB_) *P*_AC_; and (4) incorrectly retrieving both B and C is equal to (1 – *P*_AB_) × (1 – *P*_AC_).[Table-anchor tbl1]

#### Statistical analyses

For the main analysis of retrieval accuracy (proportion correct), we report a 2 × 2 × 2 (Session × Loop × Sleep) mixed ANOVA with the within-subject factor session referring to T1 (immediate) versus T2 (12-hr delay), the between-subjects factor Loop referring to whether participants encoded open- versus closed-loops, and the between-subjects factor Sleep referring to whether T1 was followed by sleep versus wakefulness. The main analysis reports memory performance for items at T2 that were not previously tested at T1. We also report a 2 × 2 × 2 (Tested × Loop × Sleep) mixed ANOVA for memory performance at T2 with the within-subject factor Tested referring to whether the triplets had previously been tested at T1 or not (reported in the online supplemental material).

For the main dependency analysis, we reported a 2 × 2 × 2 (Session × Loop × Sleep) mixed ANOVA where the dependent variable refers to the difference between the proportion of joint retrieval in the data and independent model (referred to as ‘dependency’). We also report a 2 × 2 × 2 (Tested × Loop × Sleep) mixed ANOVA of retrieval dependency at T2 with the within-subject factor tested again referring to whether triplets had previously been tested at T1 or not (reported in the online supplemental material). We also report *t* tests comparing proportion of joint retrieval in the data with their respective independent models (data vs. independent model).

Alpha was set to .05 (two-tailed) for all statistical tests. For each ANOVA, we report a partial eta-squared effect size (η_*p*_^2^). For *t* tests, we report a Cohen’s *d* as the mean difference between the condition divided by the pooled standard deviation across conditions (Lakens, 2013) as an estimate of the between-subjects effect size (regardless of whether the effect is within- or between-subjects). For the sake of consistency, when any significant effect is associated with a *p* value of > .04, or any nonsignificant effect is associated with a *p* value of < .06, we note this regardless of whether the effect is significant, nor whether the contrast is of particular theoretical interest. All statistical analyses were conducted using JASP (JASP Team, 2018).

#### Data availability

All second-level data (i.e., means per participant and condition) across all experiments for retrieval accuracy and dependency are freely available at http://osf.io/k495x/.

### Results

#### Retrieval accuracy

**Figure 3 fig3:**
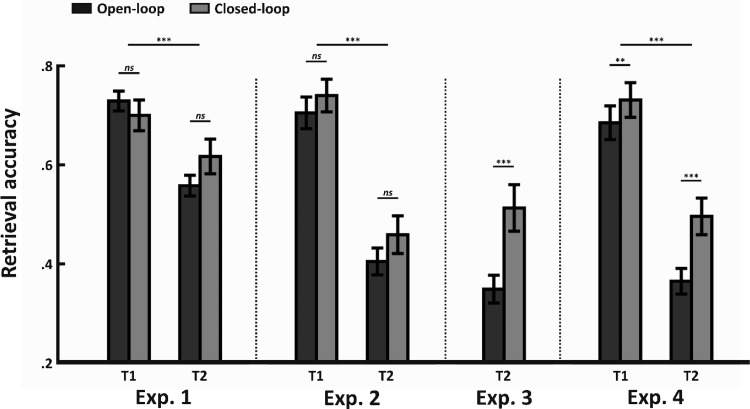
Mean proportion correct for open- and closed-loops at test sessions T1 and T2 for Experiments 1 through 4. For T2, only trials where participants retrieved events not previously tested at T1 are included. Error bars represent +/−1 standard error. Exp = Experiment. ** *p* < .01. *** *p* < .001.

Mean proportion correct (and standard deviations) across session, loop, and sleep are presented in Table 2, and mean proportion correct across loop and session (collapsed across sleep) is presented in Figure 3. Figure 3 suggests retrieval accuracy decreased over time, from T1 to T2, with perhaps more forgetting for open- than closed-loops.[Table-anchor tbl2][Fig-anchor fig3]

A 2 × 2 × 2 (Session × Loop × Sleep) ANOVA revealed a significant effect of session, with accuracy decreasing from T1 to T2, *F*(1, 100) = 352.02, *p* < .001, η_*p*_^2^ = .78. We also saw a significant interaction between session and sleep, *F*(1, 100) = 59.06, *p* < .001, η_*p*_^2^ = .37, with significantly more forgetting between T1 and T2 in the awake relative to sleep condition. Thus, we see significant forgetting across sessions that is further modulated by whether participants slept between T1 and T2. This provides a high degree of variability in performance to assess whether dependency changes as a function of forgetting, with mean retrieval accuracy ranging from .51 to .73.

No further main effects were seen for loop, *F*(1, 100) = .16, *p* = .69, η_*p*_^2^ < .01, and sleep, *F*(1, 100) = 3.88, *p* = .052, η_*p*_^2^ = .04 (though we note the borderline *p* value for the main effect of sleep). A further interaction between session and loop was also seen, *F*(1, 100) = 42.46, *p* < .001, η_*p*_^2^ = .30, revealing greater forgetting for open- than closed-loops. This interaction appeared to occur regardless of sleep, given there was no Session × Loop × Sleep interaction, *F*(1, 100) = .15, *p* = .70, η_*p*_^2^ < .01. Thus, forgetting between T1 and T2 was modulated independently by both sleep and loop.

#### Retrieval dependency

**Figure 4 fig4:**
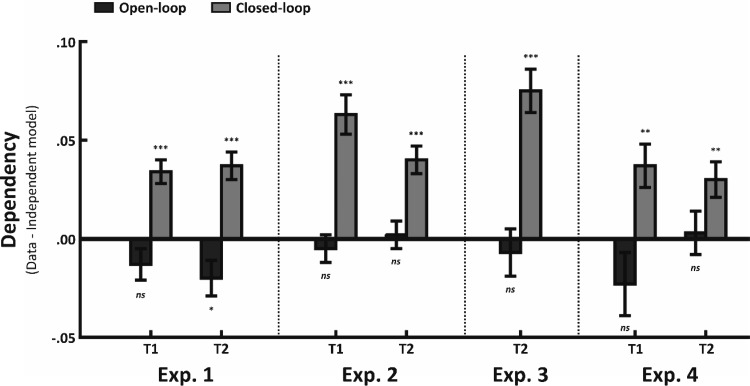
Dependency for open- and closed-loops at test sessions T1 and T2 for Experiments 1 through 4. For T2, only trials where participants retrieved events previously not tested at T1 are included. Error bars represent +/–1 standard error. Exp = Experiment. *ns* = not significant. *p < .05. ** *p* < .01. *** *p* < .001.

Mean proportion of joint retrieval (and standard deviations) for the data and independent model for open- and closed-loops, collapsed across sleep and awake conditions, are presented in Table 3 (for the means across all conditions, the data are available at http://osf.io/k495x/). Figure 4 shows the dependency across sessions and loop (collapsed across sleep and awake conditions).[Table-anchor tbl3][Fig-anchor fig4]

Consistent with previous research (Horner & Burgess, 2014), we saw no evidence of dependency for open-loops at T1, *t*(51) = 1.63, *p* = .11, *d* = .09, but dependency was seen for closed-loops, *t*(51) = 6.03, *p* < .001, *d* = .20, at T1. The critical question is what occurs at T2 given a significant proportion of the pairwise associations have been forgotten. Figure 4 shows that a similar pattern of dependency is seen at T2, with closed-loops still showing dependency, *t*(51) = 5.31, *p* < .001, *d* = .23, whereas significant antidependency was seen for open-loops, *t*(51) = 2.48, *p* = .02, *d* = .26.

Antidependency in the open-loops suggests that associations interfere with each other. It is possible that antidependency emerges during the retention period between T1 and T2 as antidependency is not observed at T1. Although no significant antidependency was seen at T1, we believe it is likely that the associations already interfere with each other either at immediate retrieval, or at the point of encoding, given that a lower proportion of joint retrieval in the data, relative to the independent model, is observed immediately after encoding. Consistent with this, the main analysis showed that dependency did not change significantly between T1 and T2 across the closed- and open-loop conditions. Note, this antidependency effect is not replicated in Experiments 2 through 4.

A 2 × 2 × 2 (Session × Loop × Sleep) ANOVA on the dependency revealed a significant main effect of loop, *F*(1, 100) = 37.02, *p* < .001, η_*p*_^2^ = .27, confirming significantly greater dependency for closed- than open-loops. We saw no evidence for changes in dependency across session, *F*(1, 100) = .25, *p* = .62, η_*p*_^2^ < .01, nor did session interact with sleep, *F*(1, 100) = .16, *p* = .70, η_*p*_^2^ < .01, or loop, *F*(1, 100) = .94, *p* = .34, η_*p*_^2^ < .01. Indeed, no other significant effects or interactions were seen (*F*s < 1.17, *p*s > .28), beyond the main effect of loop. Thus, we found no evidence to suggest that dependency was modulated by session or sleep. In sum, despite large variation in retrieval performance at T2 relative to T1 as a function of sleep (and testing; see the online supplemental material), dependency in the closed-loop and open-loop condition remained consistent across all conditions.

#### Mnemonic integration during sleep?

As outlined in the preceding text, we were also interested in assessing the possible role that sleep plays in integrating overlapping information. For this analysis, we focus solely on the open-loops as these are equivalent to the A–B A–C structures encoded in Lau et al. (2010). The main analysis in the preceding text found an overall effect of sleep on accuracy (that did not interact with loop), but no effect on dependency. Here, given our specific interest in whether sleep modulates mnemonic inference, we only report analysis for the open-loop condition (as no inference is possible for closed-loops).

For open-loop retrieval accuracy, a 2 × 2 (Session × Sleep) ANOVA revealed a significant Session × Sleep interaction, *F*(1, 50) = 25.30, *p* < .001, η_*p*_^2^ = .34, confirming the preceding analysis showing that sleep decreases forgetting for directly encoded pairs. We also assessed participants’ ability to infer nonencoded B–C pairs at T2 (see Table 4). Note, we did not assess B–C inference at T1 because this may have increased participants’ awareness of the relationship between all overlapping pairs, biasing us to finding increases in dependency for open-loops.[Table-anchor tbl4]

One participant in the sleep condition was excluded from this analysis due to a failure to respond during the inference task (missing responses >.80). Accordingly, 51 participants (26 in the awake condition, and 25 in the sleep condition) were included in the analysis. Consistent with Lau et al. (2010), we saw greater B–C inference performance in the sleep, relative to awake condition, *t*(49) = 2.03, *p* = .048, *d* = .57 (though we note the borderline *p* value). Importantly, a 2 × 2 (Session × Sleep) ANOVA failed to show any evidence for a change in dependency for open-loops between T1 and T2, *F*(1, 50) = .72, *p* = .40, η_*p*_^2^ = .01 (see Table 5), nor did session interact with sleep, *F*(1, 50) = .16, *p* = .70, η_*p*_^2^ < .01 Thus, we see evidence for increases in inference performance, but no evidence for an increase in dependency, as a function of sleep.[Table-anchor tbl5]

### Discussion

Experiment 1 modulated retrieval accuracy by manipulating: (1) the time between study and test, (2) whether participants slept between study and test, and (3) whether pairwise associations were previously tested or not (results reported in the online supplemental material). We saw evidence for effects of all three manipulations on retrieval accuracy for pairwise associations, such that across conditions we saw large variations in the amount of forgetting. Despite this, we saw no evidence for changes in dependency in either open- or closed-loops. No dependency (or antidependency) was seen for open-loops, and dependency was consistently seen for closed-loops. Experiment 1 therefore provides evidence that dependency does not change over time—closed-loops retain their dependency whereas open-loops do not show dependency.

We also saw no evidence for mnemonic integration during sleep (as measured by retrieval dependency), suggesting that the role sleep plays in increasing mnemonic inference is unlikely to be driven by encoding generalization during sleep and is more likely driven by “on-the-fly” processes at the point of retrieval; the probability of which is increased due to less forgetting for directly encoded pairs in the sleep than awake condition. Although we saw no evidence for increases in dependency for open-loops following sleep, participants were able to make the correct mnemonic inferences at a level well above chance. Our task instructions were ambiguous in relation to the inference task—that is, participants were presented with inference trials as if they were retrieval trials. Further work is needed to clarify whether participants were making correct inferences based on false memories for nonencoded pairs, or whether they were making informed inferences. However, the lack of dependency following sleep suggests that this inference process is likely to be occurring at the point of retrieval.

## Experiment 2

Despite evidence for dependency in the closed-loop condition after 12 hr in Experiment 1, we wondered whether increased forgetting might lead to decreases in dependency. Specifically, we speculated that the amount of forgetting in Experiment 1 was not sufficient to produce fragmentation, and in turn, decreases in dependency. In Experiments 2 through 4 we therefore tested participants after a week, rather than a 12-hr delay. This extended interval between T1 and T2 produced greater amounts of forgetting relative to Experiment 1, creating a sterner test for our hypothesis that event-based representations are forgotten in an all-or-none manner.

### Method

Experiment 2 was identical to Experiment 1 with the following exceptions. Experiment 2 equated to a 2 × 2 design, with the factors session and loop. No factor of sleep was included, given the interval between study and test was 1 week.

#### Participants

Fifty-two participants gave informed consent to participate in Experiment 2. Participants were recruited from the University of York student population. Participants took part in exchange for course credit or monetary compensation. Participants were allocated to one of two conditions. Twenty-six participants in the open-loop condition (23 female, *M* age = 19.46 years, age range = 18–23) and 26 participants in the closed-loop condition (23 female, *M* age = 20.00 years, age range = 18–26).

#### Procedure

In order to increase the amount of forgetting relative to Experiment 1, the two sessions were separated by one week. All sessions took place in the afternoon. Encoding and T1 took place between approximately 12 to 5 p.m. (open-loop: *M* = 2:31 p.m., range = 12:00–4:46 p.m.; closed-loop: *M* = 2:15 p.m., range = 11:58 a.m.–4:47 p.m.). T2 took place 1 week later between approximately 12 to 5 p.m. (open-loop: *M* = 2:29 p.m., range = 11:51 a.m.–4:41 p.m.; closed-loop: *M* = 2:08 p.m., range = 11:58 a.m.–4:41 p.m.). Missing responses during test (*M* = .04, *SD* = .04) were again treated as incorrect trials. There was no difference in the proportion of missed responses (collapsed across session) between open- and closed-loops, *t*(50) = .89, *p* = .38, *d* = .25.

### Results

#### Retrieval accuracy

Mean proportion correct across conditions are shown in Table 2 and Figure 3. Retrieval accuracy was .72 at T1 and .43 at T2. This is compared with retrieval accuracy of .72 at T1 and .59 at T2 in Experiment 1. Thus, increasing the interval between T1 and T2 to one week led to numerically greater forgetting relative to a 12-hr interval. A 2 × 2 (Session × Loop) ANOVA revealed a main effect of session, *F*(1, 50) = 318.83, *p* < .001, η_*p*_^2^ = .86, confirming a significant decrease in performance at T2 relative to T1. No further effects or interactions were seen (*F*s < 1.01, *p*s > .32). As such, Experiment 2 produced a significant amount of forgetting from T1 to T2, regardless of whether the triplets were encoded as open- or closed-loops.

#### Retrieval dependency

Mean proportion of joint retrieval (and standard deviations) for the data and independent model across conditions are presented in Table 3. Figure 4 shows the dependency across session and loop. As in Experiment 1, we saw no evidence for dependency for open-loops at T1, *t*(25) = .67, *p* = .51, *d* = .03, or T2, *t*(25) = .28, *p* = .78, *d* = .03, but significant evidence for dependency for closed-loops at both T1, *t*(25) = 5.90, *p* < .001, *d* = .42, and T2, *t*(25) = 5.31, *p* < .001, *d* = .51. A 2 × 2 (Session × Loop) ANOVA on dependency revealed a significant effect of loop, *F*(1, 50) = 39.96, *p* < .001, η_*p*_^2^ = .44, confirming that dependency was significantly greater in the closed- relative to open-loop condition. No interaction between session and loop was seen, *F*(1, 50) = 3.34, *p* = .07, η_*p*_^2^ = .06. In order to interrogate this marginal interaction further, we performed a *t* test between dependency at T1 and T2 separately for each loop type. Consistent with the main analyses, we saw no evidence for a change in dependency for either closed-, *t*(25) = 1.72, *p* = .10, *d* = .49, or open-loops, *t*(25) = .733, *p* = .47, *d* = .20, between T1 and T2. Critically, as shown in the preceding text, dependency was still significant in the closed-loop condition at T2. As in Experiment 1, despite high levels of forgetting between T1 and T2, we saw no evidence for a decrease in dependency between T1 and T2 for closed-loops.

### Discussion

Experiment 2 produced greater amounts of forgetting following an interval of 1 week between study and test, relative to Experiment 1. Despite this increase in forgetting, as measured by retrieval accuracy, we again saw no decrease in dependency for closed-loops, nor any increase in dependency for open-loops. Experiment 1 and 2 showed that forgetting can be affected by several post-encoding factors, such as the interval between study and test, post-encoding sleep, and retrieval practice (see the online supplemental material). However, across these factors, we find no evidence for decreases in dependency for closed-loops.

## Experiment 3

Retrieval accuracy is typically greater for closed- than open-loops when both conditions are learned within-subject (i.e., each participant learns both closed- and open-loops; Horner et al., 2015). In Experiment 1 and 2, we saw little evidence that retrieval accuracy was higher for closed- than open-loops. In Experiment 3, we aimed to assess whether the lack of difference in retrieval accuracy between closed- and open-loops in Experiments 1 and 2 was a function of the between-subjects design. This is theoretically important because if closed-loops are associated with higher accuracy relative to open-loops in a within-subject, but not between-subjects manipulation, it might suggest a possible competitive mechanism between mnemonic representations (see General Discussion). In Experiment 3, participants learned both closed- and open-loops at T1 and were tested in a single session (T2) after a week. Note that no immediate test was performed as we wanted to keep the overall number of triplets per condition consistent across Experiments 1 through 3 (30 per condition). Experiment 3 also provided a further opportunity to replicate the pattern of dependency seen for closed- and open-loops across the course of a week.

### Method

Experiment 3 was identical to Experiment 2 with the following exceptions.

#### Participants

Twenty-six participants (22 female, *M* age = 19.35, age range = 18–23) gave informed consent to participate in Experiment 3. Participants were recruited from the University of York student population. Participants took part in exchange for course credit or monetary compensation.

#### Materials

Sixty randomized location-person-objects triplets were generated for each participant. Thirty triplets were randomly assigned to the within-subject open- and closed-loop conditions, respectively.

#### Procedure

The two sessions were separated by one week. All sessions took place in the afternoon. Encoding took place between approximately 12 to 5 p.m. (*M* = 2:24 p.m., range = 11:57 a.m.–4:41 p.m.). T2 took place one week later between approximately 12 to 5 p.m. (*M* = 2:23 p.m., range = 11:56 a.m.–4:39 p.m.).

##### Encoding

Participants were presented with specific pairwise associations for each of the 60 triplets. For 30 out of the 60 triplets, participants encoded all three possible pairwise associations forming closed-loops. For the remaining 30 triplets, participants encoded two out of three possible pairwise associations forming open-loops. The encoding phase consisted of three blocks of 30, 60, and 60 trials, making a total of 150 encoding trials. During the first block, only pairwise associations for closed-loops were presented. This ensured that the duration between encoding of the last pairwise association and T2 was consistent across closed- and open-loops. In Blocks 2 and 3, the open- and closed-loops associations were presented randomly in an intermixed manner.

##### Test

No immediate test followed encoding. This was done in order to maintain consistency in the number of closed- and open-loops tested at T2 across Experiments 2 and 3. At T2, all 60 triplets were tested. Cue–target associations were presented across six blocks, making a total of 300 trials. Note, none of these cue–target associations had been tested previously at T1. As at encoding, open- and closed-loops were presented randomly within each block. As open-loops were formed of only two out of the three possible pairwise associations, the four possible cue–target associations per open-loop were randomly distributed across four out of the six blocks. Note that this necessitates that the number of trials per block can vary between participants. Missing responses (*M* = .04, *SD* = .07) were treated as incorrect trials. There was no difference between open- and closed-loops in the proportion of missing responses, *t*(25) = 1.97, *p* = .06, *d* = .16 (though we note the borderline *p* value).

#### Analysis

For retrieval accuracy, we report a paired samples *t* test comparing performance for closed- versus open-loops. For retrieval dependency, we report a paired samples *t* test comparing the proportion of joint retrieval for the data and independent model for closed- versus open-loops.

### Results

#### Retrieval accuracy

Mean proportion correct for open- and closed-loops are shown in Table 2 and Figure 3. Retrieval accuracy at T2 was .43 (averaged across open- and closed-loops). This is comparable to .43 in Experiment 2. Accordingly, we see numerically similar performance at T2 for Experiment 2 and 3. Importantly, accuracy for closed-loops (.51) was greater than open-loops (.35) following a one week delay, *t*(25) = 6.31, *p* < .001, *d* = .84. In contrast to Experiment 2, here we saw a significant difference in performance between closed- and open-loops.

#### Retrieval dependency

Consistent with Experiment 1 and Experiment 2, dependency was greater for closed- than open-loops, *t*(25) = 5.35, *p* < .001, *d* = 1.33, with closed-loops again showing significantly greater proportion of joint retrieval in the data than in the independent model, *t*(25) = 6.40, *p* < .001, *d* = .73, and open-loops showing no evidence for dependency, *t*(25) = .56, *p* = .58, *d* = .08 (see Table 3 and Figure 4).

### Discussion

Experiment 3 replicated the pattern of dependency seen in Experiments 1 and 2. We saw no dependency for open-loops and significant dependency for closed-loops. Presuming a significant amount of forgetting has occurred in Experiment 3, as seen in Experiments 1 and 2, we again showed that dependency for closed-loops is resilient to forgetting. Interestingly, we saw a significant difference in retrieval accuracy between closed- and open-loops; a pattern we did not see in Experiment 2. The critical difference between Experiments 2 and 3 is that the loop manipulation was a between-subjects factor in Experiment 2 but a within-subject factor in Experiment 3.

However, Experiment 3 did not include an immediate test (in contrast to Experiment 2). We do not know whether this difference between closed- versus open-loops in a within-subject design would also be present at T1. In other words, is the difference in performance between closed- and open-loops in Experiment 3 a result of high retrieval accuracy for closed- versus open-loops (regardless of retention interval), or are closed-loops associated with higher levels of retention over time? In Experiment 4, the loop manipulation was again a within-subject factor, however we also included an immediate, as well as delayed, test. Note, this decreased the number of triplets per condition from 30 to 15.

## Experiment 4

Experiment 4 included a test at both T1 and T2. This allowed us to see if the difference in retrieval accuracy for closed- versus open-loops at T2 in Experiment 3 was also present at T1. It also presented an opportunity to replicate the T2 retrieval accuracy difference seen in Experiment 3. Finally, Experiment 4 offered an opportunity to replicate the pattern of dependency for closed-loops seen in Experiments 1 through 3.

### Method

Experiment 4 was identical to Experiment 3 with the following exceptions.

#### Participants

Twenty-seven participants gave informed consent to participate in Experiment 4. Participants were recruited from the University of York student population. Participants took part in exchange for course credit or monetary compensation. One participant was excluded due to a failure to respond at T2 (missing responses > .50). Accordingly, 26 participants (25 female, *M* age = 19.27, age range = 18–23) were included in the analysis.

#### Procedure

Encoding took place between approximately 12 to 5 p.m. (*M* = 2:26 p.m., range = 12:02–4:56 p.m.). T2 took place one week later between approximately 12 to 5 p.m. (*M* = 2:26 p.m., range = 11:47 a.m.–16:48 p.m.). For T1, 30 out of 60 triplets were tested. Triplets were randomly assigned to the within-subject condition tested (i.e., tested at T1) versus not-tested (i.e., not tested at T1; reported in the online supplemental material). This allowed us to assess retrieval accuracy and dependency for 15 open-loops and 15 closed-loops immediately after encoding. Cue–target associations were presented across six blocks, making a total of 150 trials. A break of 20 s followed every 25 trials. At T2, all 60 triplets were tested. Cue–target associations were presented across six blocks, making a total of 300 trials. A 20 s break followed every 30 trials. Again, the four possible cue–target associations per open-loop were randomly distributed across the six blocks. We treated missing responses (*M* = .06, *SD* = .05) as incorrect trials. There was no difference between open- and closed-loops (collapsed across session) in the proportion of missing responses, *t*(25) = 1.60, *p* = .12, *d* = .20.

#### Analysis

For the main analysis of accuracy, we report a 2 × 2 (Session × Loop) within-subject ANOVA. We also report a 2 × 2 (Tested × Loop) within-subject ANOVA for retrieval accuracy at T2 (reported in the online supplemental material). For the dependency analysis, we report a 2 × 2 (Session × Loop) within-subject ANOVA where the dependent variable again refers to the difference between the proportion of joint retrieval in the data and independent model. We also report a 2 × 2 (Tested × Loop) within-subject ANOVA where the within-subject factor Tested refers to whether the triplets had previously been tested at T1 or not (reported in the online supplemental material).

### Results

#### Retrieval accuracy

Mean proportion correct across conditions are shown in Table 2 and Figure 3. Retrieval accuracy was .71 at T1 and .43 at T2. This is consistent with performance seen in Experiments 2 and 3. A 2 × 2 (Session × Loop) ANOVA revealed a main effect of session, *F*(1, 25) = 182.14, *p* < .001, η_*p*_^2^ = .88, in addition to a significant main effect of loop, *F*(1, 25) = 27.61, *p* < .001, η_*p*_^2^ = .53, with greater accuracy for closed- relative to open-loops at both T1, *t*(25) = 2.86, *p* = .008, *d* = .26, and T2, *t*(25) = 5.12, *p* < .001, *d* = .79. Interestingly, a significant Session × Loop was also observed, *F*(1, 25) = 10.40, *p* < .01, η_p_^2^ = .29, with the difference between closed- and open-loops increasing from T1 to T2. Thus, closed-loops show both higher retrieval accuracy (regardless of retention interval) and higher levels of retention relative to open-loops.

#### Retrieval dependency

Mean proportion of joint retrieval (and standard deviations) for the data and independent model across conditions are presented in Table 3. Dependency across session and loop is shown in Figure 4. Consistent with Experiments 1 through 3, we found no evidence for dependency for open-loops at T1, *t*(25) = 1.39, *p* = .18, *d* = .16, and T2, *t*(25) = .24, *p* = .81, *d* = .03. Similarly, we saw significant dependency for closed-loops at both T1, *t*(25) = 3.29, *p* < .01, *d* = .25, and T2, *t*(25) = 3.21, *p* < .01, *d* = .38. A 2 × 2 (Session × Loop) within-subject ANOVA on dependency revealed a significant main effect of Loop, *F*(1, 50) = 20.20, *p* < .001, η_*p*_^2^ = .45, with significantly greater dependency for closed- than open-loops. No other significant main effect or interaction was seen (*F*s < 1.71, *p*s > .20). Consistent with Experiments 1 through 3, closed-loops retain their dependency despite high levels of forgetting.

### Discussion

Experiment 4 replicated Experiments 1 through 3, showing consistent dependency for closed-loops despite high levels of forgetting. Experiment 4 showed higher retrieval accuracy for closed- than open-loops at T2, consistent with Experiment 3. This accuracy difference was present at T1 (consistent with the results of Horner et al., 2015), however the effect was significantly greater after a week. This presents evidence that the structure of overlapping associations can affect long-term retention, but seemingly only when structures are manipulated in a within-subject design (as in Experiments 3 and 4, relative to Experiment 2). We return to this finding in the General Discussion.

## A Model of Independent Forgetting

Across four experiments we provide evidence for consistent levels of retrieval dependency, despite varying levels of forgetting. We consistently saw evidence for dependency for closed-loops at both T1 and T2 and, importantly, we saw that dependency was retained despite variable levels of forgetting. If complex events fragment as a function of forgetting, such that some aspects of the memory trace are forgotten more quickly than others (e.g., *kitchen* is forgotten, but not *hammer* or *Barack Obama*), then we would expect to see a decrease in dependency over time.

To ensure that the levels of dependency seen at T2 across Experiments 1 through 4 were greater than expected if forgetting was independent, we created a new model of independent forgetting. The independent model used in the main analyses predicts the level of dependency if the retrieval of associations for a given event are independent. It takes into account each participant’s retrieval accuracy at each time point (separately) but does not take into account the amount of forgetting for each participant. Accordingly, we created a model that predicted the level of dependency at T2, given a participant’s retrieval accuracy and rate of forgetting between T1 and T2. The model can therefore be thought of as a model of independent forgetting, as opposed to a model of independent retrieval. It predicts the level of dependency expected at T2 if events are forgotten in an independent manner.

We simulated individuals’ retrieval data across all events and pairwise associations at T2 based on their performance at T1 and their overall level of forgetting (i.e., the difference in retrieval accuracy between T1 and T2). Note, the model includes a single forgetting parameter, such that the mean rate of forgetting is (on average) uniform across all events and element-types. The simulated data was based on the assumption of independent forgetting, such that forgetting of one pairwise association for an event was not predictive of forgetting for any other pairwise association for that event. Specifically, we took performance for each T1 retrieval trial, across all cue-test pairs, which resulted in a 6 × *N* matrix (with six cue-test pairs, and *N* events) where each trial was either correct or incorrect. We then simulated performance at T2 by converting correct trials to incorrect trials randomly until mean performance for the 6 × *N* matrix was equated to observed performance at T2. Importantly, each correct trial at T1 had a probability of being simulated as incorrect at T2 based on the mean level of forgetting for that participant. We then calculated the level of retrieval dependency for this simulated data set. This gives us the level of dependency for an individual participant at T2 under the assumption of independent forgetting. If the dependency seen in the observed data is greater than the simulated data, then this provides positive evidence that forgetting of the pairwise associations for a complex event does not occur in an independent manner. For each participant, we simulated 100 data sets, and present the mean dependency from across these simulations. Because Experiment 3 did not include an immediate test, we could only assess independent forgetting in Experiments 1, 2, and 4. We focused our modeling solely on closed-loops, given this is the condition that shows dependency at T1 and T2.

### Results

Mean proportion of joint retrieval (and standard deviations) for the data and independent model for the simulated and observed data at T2 are presented in Table 6. We first asked whether dependency for closed-loops in the simulated data at T2 showed a decrease relative to observed dependency at T1. In other words, if forgetting was independent, does our model predict a decrease in dependency between T1 and T2?[Table-anchor tbl6]

We first report a 2 × 3 (Session × Experiment) ANOVA where the within-subject factor Session refers to the observed dependency at T1 versus the simulated dependency at T2 and the between-subjects factor Experiment refers to Experiment 1, 2, and 4. This ANOVA revealed a significant main effect of Session, *F*(1, 101) = 61.91, *p* < .001, η_*p*_^2^ = .38, with dependency decreasing between T1 and T2. A Session × Experiment interaction was also seen, *F*(2, 101) = 11.77, *p* < .001, η_*p*_^2^ = .19, with a significantly greater decrease in dependency between T1 and T2 in Experiment 2 relative to Experiment 1, *t*(25) = 3.52, *p* < .01, *d* = 1.09, and Experiment 4, *t*(25) = 2.18, *p* = .04, *d* = .71. Our model of independent forgetting therefore predicts a significant decrease in dependency between T1 and T2 for closed-loops. No such decrease was seen in the observed dependency in Experiments 1, 2, and 4.

We also performed a Bayesian pairwise *t* test comparing observed dependency at T1 with simulated dependency at T2 across all participants from Experiments 1, 2, and 4 (*N* = 104). The Bayes factor was >1,000 in favor of the hypothesis that dependency should decrease as a function of independent forgetting (exceedance probability > .99; prior Cauchy distribution *r* = .707, centered at 0; null hypothesis = no decrease in dependency between T1 and T2). This provides strong evidence that dependency should decrease if closed-loops fragment as a function of forgetting.

We next asked whether we saw greater dependency for closed-loops in the observed data at T2 relative to the simulated data at T2. This analysis tells us whether the observed dependency at T2 is greater than that predicted by the independent model of forgetting. We report a 2 × 3 (Model × Experiment) ANOVA with the within-subject factor Model referring to observed dependency at T2 versus the simulated dependency at T2. Here we saw a significant main effect of Model, *F*(1, 101) = 10.43, *p* < .01, η_*p*_^2^ = .09, with greater dependency in the observed relative to the simulated data.

Finally, we performed a Bayesian pairwise *t* test comparing observed dependency at T1 with observed dependency at T2 across all participants from Experiments 1, 2, and 4 (*N* = 104). The Bayes factor = 5.41 in favor of the null hypothesis that dependency does not decrease as a function of forgetting (exceedance probability = .84; prior Cauchy distribution *r* = .707, centered at 0; null hypothesis = no decrease in dependency between T1 and T2), provides positive evidence that dependency does not decrease over time.

### Discussion

The independent forgetting model estimates dependency at T2 under the assumption that cue–target associations within an event are independently forgotten. Compared with the observed dependency, we show that independent forgetting of individual event elements predicts a significant decrease in dependency between T1 and T2, implying a fragmentation of the underlying memory trace. Critically, we also saw significantly greater dependency in the observed data at T2 relative to the simulated data. A Bayesian analysis also provided positive evidence that the observed dependency does not decrease as a function of forgetting for closed-loops.

## General Discussion

Across four experiments, we provide consistent evidence for retrieval dependency for closed-loops after a delay, despite variable levels of forgetting. We also show that retrieval dependency does not change for open-loops; they do not show retrieval dependency immediately after encoding, nor after a delay. Further, we developed a model of independent forgetting, providing evidence for levels of dependency for closed-loops that are greater than that predicted under an assumption of independent forgetting. Together, we take these findings to support our hypothesis that coherent (closed-loop) event representations tend to be forgotten in an all-or-none manner, with closed-loops being more likely to either be retained or forgotten in their entirety.

In Experiment 1, we showed that dependency for closed-loops is retained across a 12-hr interval, irrespective of whether T1 is followed by sleep or wakefulness. Consistent with previous findings (e.g., Barrett & Ekstrand, 1972; Gais, Lucas, & Born, 2006; Jenkins & Dallenbach, 1924; Lahl, Wispel, Willigens, & Pietrowsky, 2008; Plihal & Born, 1997; Tucker et al., 2006), we showed that sleep reduces forgetting, but does not change the form that forgetting takes. Experiment 1 also provided an opportunity to directly assess the extent to which sleep (relative to being awake) supports the integration of overlapping information (Lau et al., 2010). We found no evidence that sleep promotes integration of open-loops, but it does appear to improve our ability to make inferences between related information (i.e., inferring that B–C items are related after directly encoding A–B and A–C pairs).

In Experiment 2, we increased the interval between study and test to 1 week, increasing overall levels of forgetting. Despite this increase in forgetting, we saw no evidence for changes in dependency for closed- or open-loops. Experiments 3 and 4 replicated Experiment 2, providing further evidence for dependency for closed-loops when the interval between study and test was 1 week. We therefore provide consistent evidence that forgetting is not associated with decreases in dependency for closed-loop events.

Experiments 3 and 4 also showed that the structure of the underlying mnemonic representation can support both immediate and long-term retention. Retrieval accuracy was higher for closed- than open-loops, a difference that increased significantly over the course of a week. Interestingly, this effect was only seen in a within-subject design where each participant learned both closed- and open-loops (in Experiments 3 and 4), but not in a between-subjects design where each participant either learned closed- or open-loops (in Experiment 2).

The results presented here have implications for (1) how coherent event representations are forgotten, (2) whether sleep promotes the integration of overlapping information, and (3) how associative structure can boost retention of information in the long-term. We discuss each of these topics in the following text.

### Forgetting of Coherent Event Representations

Despite a long-standing interest in forgetting, little research has focused on how memory representations change as a function of forgetting. Here we used the presence of dependency to infer the coherence of an underlying memory trace and asked how dependency changes as a function of forgetting. A similar approach was used by Brady et al. (2013), where they assessed dependency for specific properties of an object (i.e., “exemplar” and “state”). They found that dependency decreased over time, such that the exemplar (e.g., shape of a glass) and state (e.g., contents of the glass) of an object were forgotten independently. Thus, object- or item-based representations appear to fragment over time. This result appears at odds with the current results where we see no evidence for a decrease in dependency for closed-loops over time. However, here we were specifically interested in forgetting of coherent episodic events that require the multimodal binding of three distinct elements or items—that is, a location, person, and object. Thus, whereas Brady et al. (2013) focused on item-based representations, specifically multiple aspects of an individual item, we were interested in event-based representations, specifically associations between multiple distinct items or elements.

Neuropsychology and neuroimaging studies have demonstrated a functional dissociation between regions of the medial temporal lobe, with the perirhinal cortex supporting item-based representations and the hippocampus supporting event-based representations (Barense et al., 2005; Davachi, Mitchell, & Wagner, 2003; Diana, Yonelinas, & Ranganath, 2010; Lee et al., 2005; Ranganath et al., 2004). Although the perirhinal cortex has been implicated in certain associative processes (Mayes et al., 2004; Mayes, Montaldi, & Migo, 2007) and conjunctive representations (Barense, Gaffan, & Graham, 2007; Bussey & Saksida, 2007) of items/objects, such regions are not thought to support associations between multimodal representations (Diana et al., 2007; Eichenbaum, Yonelinas, & Ranganath, 2007). Instead, it is the hippocampus that has been implicated in the multimodal binding that is required to form more complex event representations (Cohen et al., 1999; Damasio, 1989; Davachi, 2006; Eichenbaum et al., 2007; Horner et al., 2012). The imagery task and memoranda used here were designed to require cross-modal binding (Horner et al., 2015; Horner & Burgess, 2013, 2014) given that the hippocampus has been shown to act as a convergence zone (Backus, Bosch, Ekman, Grabovetsky, & Doeller, 2016) binding multimodal information into coherent event representations (Damasio, 1989; Marr, 1971; Teyler & DiScenna, 1986). Thus, it is possible that the differences in forgetting seen between the present studies and Brady et al. (2013) relate to this dissociation between item-based perirhinal representations and event-based hippocampal representations.

This dissociation is also apparent in the psychological literature in relation to retrieval dependency. First, although Brady et al. (2013) saw dependency between the retrieval successes of exemplar and state information immediately after encoding, they provided evidence that such dependency might be primarily driven by encoding-related factors. Horner and Burgess (2014) provided evidence against an encoding-based explanation of dependency for more complex events by separating out the encoding of pairwise associations for three element events—presenting each pairwise association in separate encoding trials (as in the present studies). No difference in dependency was seen between this ‘separated’ encoding condition relative to when all three elements were encoded on a single trial (Horner & Burgess, 2014). This suggests that, even when tested immediately after encoding, the dependency seen for item-based and event-based representations might be driven by different factors. In the case of item-based representations; encoding-related factors such as attention, and in the case of event-based representations; perhaps a retrieval-related process that allows for holistic retrieval.

Evidence from the long-term source-memory literature also suggests that there is a degree of asymmetry in relation to how source details are bound to items, with information about color and location being directly, but independently, bound to item information, but not each other (Starns & Hicks, 2005, 2008; see Hicks & Starns, 2015 for a review). This lack of coherency and symmetry in item-based representations might underlie the decreases in dependency seen over time in Brady et al. (2013). As distinct aspects of an item are stored in a relatively independent manner, they are likely to also be forgotten in a similarly independent manner. In contrast, event-based representations are more coherent and symmetrical in nature (Horner et al., 2015; Horner & Burgess, 2013) and as such the forgetting of elements from a given event are more likely to be related. Interestingly, Sekeres and colleagues (2016) recently showed that ‘peripheral’ details from event-based memories are forgotten more rapidly than central details. We believe that this difference in forgetting rates for peripheral and central details might relate to the differences in the pattern of forgetting for event- and item-based memories observed here and in Brady et al. (2013), with central details of event-based memories (e.g., *Barack Obama* in the *kitchen* with a *hammer*) being more likely to be forgotten in an all-or-none manner, and peripheral/item-based details of event-based memories (e.g., the color vs. shape of *Barack Obama’s* tie) being forgotten in an independent manner.

It has recently been proposed that item-based representations that rely on the perirhinal cortex are more likely to be forgotten as a result of interference (as opposed to decay; Sadeh et al., 2014). This is because the neural representations for items in the perirhinal cortex are likely distributed and overlapping in nature. Thus, encoding similar objects results in interference due to their representational overlap. However, event-based representations are thought to be encoded in the hippocampus, where a pattern separation process supported by the dentate gyrus (Bakker, Kirwan, Miller, & Stark, 2008; Berron et al., 2016; Leutgeb, Leutgeb, Moser, & Moser, 2007; Neunuebel & Knierim, 2014) and more sparse representations (Barnes, McNaughton, Mizumori, Leonard, & Lin, 1990; Viskontas, Knowlton, Steinmetz, & Fried, 2006) are likely to reduce representational overlap between similar events. This decrease in representational overlap decreases the likelihood of interference (McClelland et al., 1995). Instead, forgetting for hippocampal event-based representations is thought to be a result of decay (Hardt et al., 2013).

Sadeh et al. (2016) provided behavioral evidence for this proposed dissociation, showing that whereas recollection (a process supported by hippocampal representations) decreased as a function of time between study and test (consistent with forgetting via decay), familiarity (a process supported by perirhinal representations) decreased as a function of experimentally induced interference. Recent evidence using “precision” measures for assessing object-color memory has shown that encoding similarly colored objects causes interference, leading to a decrease in precision—that is, participants can still remember the color, but with less specificity than previously (Sun et al., 2017). This again supports the notion that forgetting for item-based representations is more likely driven by interference than decay.

The lack of a decrease in dependency can be taken as support for the hypothesis that coherent (closed-loop) events tend to be forgotten in an all-or-none manner. If hippocampal event-based representations were forgotten by a process of decay, as proposed by Sadeh et al. (2014), this would suggest that decay is relatively uniform across the separate elements of an event. Although there is variation in the amount of decay across events, there would be less variation within an event. An alternative decay account would predict that variation is present in the rate of decay within an event, however the process of retrieval compensates for this variation. We have previously shown that closed-loops are supported by the hippocampus, and retrieved by a process of pattern completion (Horner et al., 2015; see Horner & Doeller, 2017 for a review). Pattern completion allows for the retrieval of a complete memory trace (i.e., pattern) in the presence of a partial or ambiguous input (Gardner-Medwin, 1976; Hopfield, 1982; Marr, 1971; McClelland et al., 1995; Treves & Rolls, 1992, 1994). Here, activation of a single event element (e.g., a location) triggers the reactivation of all other elements for that event (i.e., a person and an object). Under such an account, decay could be nonuniform within an event, but pattern completion in hippocampal subfield CA3 (Hopfield, 1982; Treves & Rolls, 1992), or more widespread recurrency within the hippocampal complex (Kumaran & McClelland, 2012), would lead to the presence of dependency as long as the associations between some elements are sufficiently strong. However, when decay is sufficient, the remaining associations may no longer be able to support retrieval, meaning that the entire trace cannot be retrieved. In other words, although decay might be nonuniform within an event, pattern completion produces the appearance of uniformity at retrieval, driving behavioral dependency. This same mechanism has been used to account for retrieval generalization on paired-associate tasks (e.g., where participants make inference judgments about two overlapping associations; Banino et al., 2016).

Our results do not rule out the possibility that interference (not decay) is the primary driver of forgetting for event-based representations, although this is at odds with recent theoretical and empirical work (Sadeh et al., 2014, 2016). If so, interference would need to be uniform in manner. For example, if you encoded two events that share a common location; for example, “*kitchen–Obama–hammer*” and “*kitchen-Beckham-telephone*,” the encoding of the second event involving David Beckham would have to interfere with not only retrieval success for the *kitchen–Obama* and *kitchen–hammer* associations, but also the *Obama–hammer* association—otherwise a decrease in dependency would likely be seen. Though we cannot rule out an interference account, it is not immediately clear how such uniformity could be achieved.

Although the precise mechanism that underlies the pattern of forgetting seen in the present studies is unclear, the results (taken alongside those of Brady et al. (2013)) suggest that forgetting for event-based representations is driven by a different mechanism than for item-based representations. We believe this is likely to be hippocampal-based and supported by the known recurrent circuitry in this region that has been shown to support the computational process of pattern completion. Further, this is most likely a result of decay, perhaps driven by neurogenesis of hippocampal granule cells (Frankland et al., 2013), or more active regulatory changes (Hardt et al., 2013). Note that the argument here is that forgetting occurs due to a failure in pattern completion (Frankland et al., 2013), rather than an erasure of memory per se. It is entirely possible that aspects of some events, or entire events, can spontaneously recover and be brought to mind at some later point (Tulving & Pearlstone, 1966), or could even be recovered via optogenetic induction (Roy et al., 2016).

### Sleep and Mnemonic Integration

Sleep is thought to not only play an active role in the strengthening of memory traces, but also in the integration of overlapping information (Lewis & Durrant, 2011; Stickgold & Walker, 2013). For example, Lau et al. (2010) presented evidence for an increase in participant’s ability to infer the relationship between A–C pairs following a nap after directly encoding A–B and B–C pairs. This increase in inference ability postnap was taken as evidence for sleep playing an active role in the integration of A–B and B–C pairs, similar to the evidence for integration seen during repeated presentations of such pairs (Zeithamova et al., 2012). However, it has recently been suggested that A–C inference can readily be supported by retrieval-related processes, supported by the recurrent connections in the hippocampus (Kumaran & McClelland, 2012). Here, the relationship between A and C is generated “on-the-fly,” via the retrieval of A–B and then B–C. Under this account, the probability of successful inference increases via increases in the associative strength of the directly encoded A–B and B–C pairs.

Here we used retrieval dependency to distinguish between these two accounts. In Experiment 1, participants learned open-loops (A–B, B–C associative structures). After sleep, we tested retrieval performance for the directly encoded pairs, and inference for the A–C nonencoded pairs. Consistent with Lau et al. (2010), we found evidence that sleep increased performance on an A–C inference task (though the effect was relatively small). However, we saw no increase in dependency as a function of sleep for open-loops. We also saw no evidence for increases in dependency for open-loops after a 1-week delay (Experiments 2 through 4), that included multiple sleep–wake cycles. If sleep does play a role in mnemonic inference, this effect is likely to be primarily driven by increases in associative strength for directly encoded pairs that allows for inference at the point of retrieval, rather than a more active sleep-related integration process.

For both open- and closed-loops, sleep decreased forgetting relative to wakefulness across a 12-hr delay (Experiment 1). Sleep appears to decrease forgetting but does not change the form that forgetting takes. This is consistent with existing models of consolidation (Frankland & Bontempi, 2005; McClelland et al., 1995; Nadel & Moscovitch, 1997; Squire & Alvarez, 1995) where sleep reduces forgetting by stabilizing existing connections between the hippocampus and neocortex, perhaps counteracting memory decay within the hippocampus (Frankland et al., 2013). However, this process appears to occur without altering the form that forgetting takes. Interestingly, we also showed that retrieval practice diminished the effect of sleep on memory (see the online supplemental material), in line with the recent proposal that retrieval practice might drive a rapid on-line consolidation process (Antony, Ferreira, Norman, & Wimber, 2017), mitigating the role of sleep (Kornell, Bjork, & Garcia, 2011).

### Closed-Loops as a Mnemonic Aid?

In Experiments 3 and 4, we saw greater retrieval accuracy for closed- than open-loops, a difference that increased over time. Indeed, after a week the difference in memory performance was substantial (.16 in Experiment 3 and .13 in Experiment 4; compared with .04 at T1 in Experiment 4). Thus, the associative structure formed across separate encoding trials appears to significantly modulate the extent of forgetting over the course of a week. This raises the possibility that learning associations between three elements in a closed-loop structure might aid long-term retention of such associations. Could associative structure at encoding be used as an educational tool, similar in nature to known mnemonic techniques such as retrieval practice (Roediger & Karpicke, 2006) and the spacing effect (Ebbinghaus, 1913)?

This proposal would be premature, given the differences seen between closed- and open-loops were only seen in Experiments 3 and 4. No difference in retrieval accuracy between closed- and open-loops was seen after a week in Experiment 2. The key difference between these experiments is the between- versus within-subject experimental design. Whereas in Experiment 2 participants either learned closed- or open-loops, in Experiments 3 and 4 participants learned both closed- and open-loops. Importantly, overall retrieval accuracy after a week (averaged across closed- and open-loops) in Experiments 3 (.43) and 4 (.44) was similar to Experiment 2 (.43). Thus, overall forgetting rates were comparable, but learning both closed- and open-loop biased forgetting such that open-loops were more likely to be forgotten than closed-loops.

One possible explanation for this effect is a competitive model of forgetting, where multiple associative structures compete for survival. Closed-loops are already associated with significantly higher retrieval accuracy at immediate test (though the numerical size of the effect is relatively small). If this higher retrieval accuracy relates to greater associative strength, then perhaps this allows closed-loops to “out-compete” open-loops to survive. Another possibility is that the coherent nature of closed-loops, that allows for pattern completion at retrieval, increases the probability that such representations are replayed during offline consolidation processes (Lewis & Durrant, 2011), increasing retrieval accuracy relative to those nonreplayed representations (i.e., open-loops). Note that these explanations are not mutually exclusive. Importantly, Poulton (1982) has argued that within-subject designs, in contrast to between-subjects designs, can bias performance (in unknown ways) due to asymmetries in encoding and/or retrieval strategies when conditions are interleaved randomly. Accordingly, the difference in retrieval accuracy between closed- and open-loops observed here could be due to the transfer of a particular strategy that is appropriate in one condition, but not in the other. Further work is needed to determine the precise experimental conditions under which closed-loops are more likely to be retained. However, if one accepts that some amount of forgetting is inevitable in any educational setting, learning a certain amount of information in a ‘closed-loop’ format might promote long-term retention of important information. Critically, if such a technique were to be used as an educational ‘tool’ to increase long-term retention, similar effects would need to be shown in developmental populations, given the protracted development of the hippocampus in childhood (Olson & Newcombe, 2013).

## Conclusion

Across four experiments, we provide consistent evidence that retrieval dependency does not change over time, despite variation in the overall amount of forgetting: The associative structure formed at encoding has a consistent, lasting, impact on the coherency of retrieval. In relation to closed-loops, the results support our hypothesis that coherent event representations tend to be forgotten in an all-or-none manner, such that events are more likely to be either forgotten or retained in their entirety. Consistent with this, we provide evidence against the notion that coherent event representations fragment as a function of forgetting by the creation of an independent model of forgetting (as opposed to the independent model of retrieval used in previous studies (Horner & Burgess, 2013, 2014). We also saw evidence that the associative structure at encoding can, under specific conditions, modulate the overall amount of forgetting. When participants learned closed- and open-loops, forgetting rates were significantly lower for closed-loops (this was not the case when participants learned either closed-loops or open-loops). Thus, we also provide evidence that retrieval accuracy and dependency can be modulated by the associative structure at encoding.

## Supplementary Material

10.1037/xge0000648.supp

## Figures and Tables

**Table 1 tbl1:** Contingency Table for the Independent Model for Correct and Incorrect Retrieval, Over N Events, for Elements B and C When Cued by A

Retrieval of Element C	Retrieval of Element B	
	Correct (*P*_AB_)	Incorrect (1 – *P*_AB_)
Correct (*P*_AC_)	$\sum_{i=1}^{N}P_{AB_{i}}P_{AC_{i}}$	$\sum_{i=1}^{N}P_{AC_{i}}(1-P_{AB_{i}})$
Incorrect (1 − *P*_AC_)	$\sum_{i=1}^{N}P_{AB_{i}}\left(1-P_{AC_{i}}\right)$	$\sum_{i=1}^{N}\left(1-P_{AB_{i}})(1-P_{AC_{i}}\right)$
*Note.* *i* = 1 to *N*.		

**Table 2 tbl2:** Mean Proportion Correct (and Standard Deviations in Parentheses) at Test Sessions at T1 and T2 for Experiments 1 Through 4

Experiment	Loop	Session	
		T1	T2
Experiment 1			
Sleep condition	Open	.73 (.16)	.61 (.16)
	Closed	.72 (.25)	.69 (.27)
Awake condition	Open	.73 (.14)	.51 (.14)
	Closed	.68 (.21)	.54 (.23)
Experiment 2	Open	.71 (.17)	.41 (.14)
	Closed	.74 (.17)	.46 (.20)
Experiment 3	Open	n/a	.35 (.15)
	Closed	n/a	.51 (.24)
Experiment 4	Open	.69 (.18)	.37 (.14)
	Closed	.73 (.18)	.50 (.19)
*Note.* For T2, only trials where participants retrieved cue–target associations not previously tested at T1 are included. n/a = not applicable.			

**Table 3 tbl3:** Mean Proportion of Joint Retrieval (and Standard Deviations in Parentheses) for the Data and Independent Model for Test Sessions at T1 and T2 for Experiments 1 Through 4

Experiment	Loop	T1		T2	
		Data	Independent	Data	Independent
Experiment 1	Open	.62 (.14)	.64 (.12)	.52 (.10)	.55 (.06)
	Closed	.71 (.17)	.68 (.18)	.69 (.15)	.66 (.17)
Experiment 2	Open	.60 (.16)	.61 (.15)	.56 (.08)	.55 (.08)
	Closed	.73 (.14)	.67 (.16)	.62 (.08)	.58 (.09)
Experiment 3	Open	n/a	n/a	.57 (.10)	.58 (.08)
	Closed	n/a	n/a	.69 (.09)	.61 (.11)
Experiment 4	Open	.57 (.16)	.59 (.12)	.56 (.10)	.56 (.08)
	Closed	.70 (.15)	.66 (.16)	.60 (.09)	.57 (.07)
*Note.* For Experiment 1, the proportion of joint retrieval is collapsed across the sleep and awake conditions. For T2, only trials where participants retrieved cue–target associations not previously tested at T1 are included. n/a = not applicable.					

**Table 4 tbl4:** Mean Proportion Correct (and Standard Deviations in Parentheses) for Encoded Cue–Target Associations at T1 and T2, and Nonencoded Pairs at T2 for the Open-Loop Condition in Experiment 1

Condition	Encoded		Nonencoded
	T1	T2	T2
Sleep	.73 (.16)	.61 (.16)	.53 (.22)
Awake	.73 (.14)	.51 (.14)	.42 (.29)
*Note.* For T2, only trials where participants retrieved cue–target associations not previously tested at T1 are included.			

**Table 5 tbl5:** Mean Proportion of Joint Retrieval (and Standard Deviations in Parentheses) for the Data and Independent Model for Test Sessions T1 and T2 for the Open-Loop Condition in Experiment 1

Condition	T1		T2	
	Data	Independent	Data	Independent
Sleep	.62 (.14)	.64 (.11)	.54 (.10)	.56 (.07)
Awake	.62 (.15)	.63 (.14)	.51 (.09)	.53 (.05)
*Note.* For T2, only trials where participants retrieved cue–target associations not previously tested at T1 are included.				

**Table 6 tbl6:** Mean Proportion of Joint Retrieval (and Standard Deviations in Parentheses) for the Data and Independent Model at T2 for the Simulated and Observed Data for Experiments 1, 2, and 4

Experiment	Simulated		Observed	
	Data	Independent	Data	Independent
Experiment 1	.68 (.16)	.65 (.17)	.69 (.15)	.66 (.17)
Experiment 2	.61 (.08)	.60 (.09)	.62 (.08)	.58 (.09)
Experiment 4	.58 (.08)	.57 (.07)	.60 (.09)	.57 (.07)
*Note.* For Experiment 1, the proportion of joint retrieval is collapsed across the sleep and awake condition. Only trials, where participants retrieved cue–target associations not previously tested at T1 are included.				

**Figure 1 fig1:**
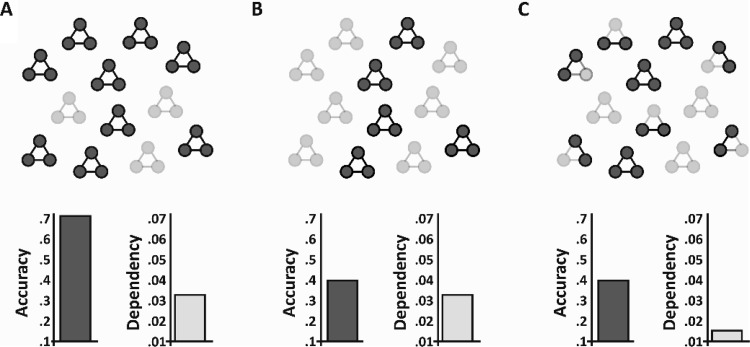
Illustration of retrieval accuracy and dependency for three-element events immediately after encoding and after all-or-none or independent forgetting has occurred. Panel A: After encoding. We assume that some events are either not encoded or are forgotten between encoding and immediate test (represented by transparent events). Retrieval is all-or-none for remembered events. All-or-none retrieval is reflected in values of dependency significantly greater than 0. Panel B: After all-or-none forgetting. Events are forgotten in an all-or-none manner. Despite decreases in retrieval accuracy, due to forgetting, dependency does not decrease relative to dependency in Panel A. Panel C: After independent forgetting. Individual associations are remembered and/or forgotten within the same event. Dependency decreases relative to dependency in Panel A, despite the same decrease in accuracy as in Panel B. Associative structures illustrate three-element events.

**Figure 2 fig2:**
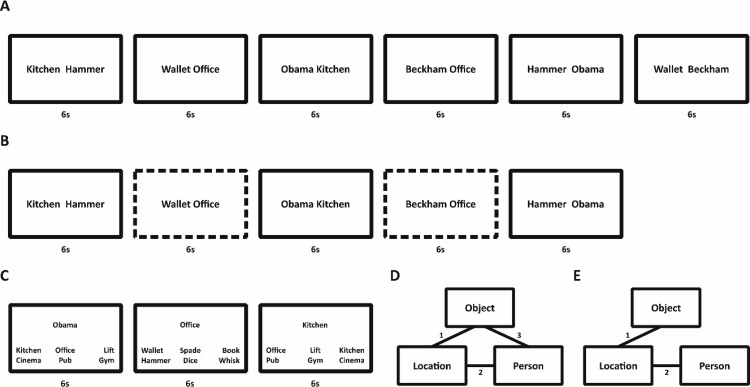
General experimental design. Panels A and B: Encoding. Participants saw multiple pairwise associations. They imagined each association interaction in “a meaningful way as vividly as possible” for 6 s. Each association was preceded by a 500-ms fixation cross and followed by a 500-ms blank screen (Panel A) Experiments 1 and 2. Participants encoded two or three overlapping pairwise associations depending on whether they were allocated to the between-subjects open- versus closed-loop conditions, respectively. In the open-loop condition, participants did not encode the third and final association (e.g., *hammer*–*Obama* and *wallet*–*Beckham*; see Panel E). Panel B: Experiments 3 and 4. Participants encoded open- and closed-loop pairwise associations in an intermixed manner. Solid and dotted lines were not presented but highlight closed- (solid lines) and open-loops (dotted lines). Panel C: Test. Participants were presented with a single cue and required to retrieve one of the other elements from the same event from among five foils (elements of the same type from other events) in 6 s. Each cued-recognition trial was preceded by a 500-ms fixation cross and followed by a 500-ms blank screen. Panel D: The associative structure of closed-loops with example encoding order for three pairwise associations (numbers 1 through 3). Panel D: The associative structure of open-loops with example encoding order for the two pairwise associations (numbers 1 and 2). The third and final associations (i.e., person-object in this example) is not shown to the participants.
