# Supplementary material for: United We Fall: All-or-None Forgetting of Complex Episodic Events
Source: J Exp Psychol Gen. 2019 Jul 15;149(2):230–48. doi: 10.1037/xge0000648 (PMC6951107; doi:10.1037/xge0000648)
Supplement: Supplementary file 1 [file xge0000648SuppMat.pdf]

# Supplementary Material

## Experiment 1

### Ratings of sleepiness

Mean sleepiness ratings (and standard deviations) are presented in Table S1. Ratings were not collected from four participants in the sleep, open-loop condition and one participant in the sleep, closed-loop condition.

**Table S1** Mean sleepiness ratings (and standard deviations) for test sessions T1 and T2 for Experiment 1 for the sleep and awake condition. Note, the Stanford Sleepiness Scale ranges from 1 (feeling active, vital, alert, or wide awake) to 7 (no longer fighting sleep, sleep onset soon, having dream-like thoughts).

| | | **Session** | |
|---|---|---|---|
| **Condition** | **Loop** | **T1** | **T2** |
| Sleep | Open | 2.23 (0.61) | 2.23 (1.07) |
| - | Closed | 3.00 (1.00) | 2.64 (1.04) |
| Awake | Open | 2.92 (1.23) | 2.23 (1.14) |
| - | Closed | 2.39 (0.80) | 2.50 (1.36) |

A 2x2x2 (Session x Loop x Sleep) mixed ANOVA showed a significant between-subject interaction between Sleep and Loop, $F(1,95) = 5.35$, $p = .02$, $\eta_p^2 = .05$, and a significant three-way interaction between Session, Sleep and Loop, $F(1,95) = 4.03$, $p = .048$, $\eta_p^2 = .04$. This three-way interaction is primarily driven by unexpected high sleepiness ratings in the sleep closed-loop condition at T1. This is unlikely to have driven our dependency results given dependency was consistent across Session. Further, none of the post hoc comparisons were significant when corrected for multiple comparisons. Thus, possible differences in sleepiness across conditions is unlikely to have contributed to our main results.

### Retrieval accuracy across element-type

We have previously demonstrated that dependency leads to greater symmetry in retrieval across the different element-types (Horner & Burgess, 2014). To assess for differences in retrieval accuracy that are due to variations in the memorability of certain modality-specific elements, we performed a 2x2x3 (Session x Loop x Element) ANOVA, collapsed across the sleep and awake condition, with the within-subject factor Element referring to the three different elements (i.e., people, locations, and objects). We performed this ANOVA separately for

instances where the element-types acted as a common cue (i.e., Cue-Element) and common retrieval target (i.e., Retrieval-Element). Table S2 provides mean proportion correct (and standard deviations) across modality-specific elements (i.e., people, locations, and objects) for the common cue and common retrieval target instance at T1 and T2 for Experiment 1 for open- and closed-loops.

**Table S2** Mean proportion correct (and standard deviations) across element-types for test sessions T1 and T2 for Experiment 1 for open- and closed-loops. Proportion correct is collapsed across the sleep and awake conditions. For T2, only trials where participants retrieved cue-target associations not previously tested at T1 are included. Element-types (i.e., people, locations, and objects) refer to the type of common cue and retrieval target element (i.e., Direction)

| | | **Session** | | | | | |
|---|---|---|---|---|---|---|---|
| | | **T1** | | | **T2** | | |
| **Direction** | **Loop** | **People** | **Locations** | **Objects** | **People** | **Locations** | **Objects** |
| Cue | Open | .73 (.16) | .74 (.16) | .72 (.15) | .55 (.17) | .59 (.18) | .54 (.16) |
| - | Closed | .69 (.24) | .72 (.23) | .69 (.23) | .60 (.27) | .62 (.25) | .62 (.26) |
| Target | Open | .74 (.16) | .74 (.15) | .71 (.15) | .56 (.16) | .56 (.17) | .56 (.17) |
| - | Closed | .71 (.23) | .69 (.23) | .70 (.23) | .61 (.27) | .62 (.26) | .62 (.25) |

Consistent with the main analysis for Experiment 1, a 2x2x3 (Session x Loop x Retrieval-Element) ANOVA, collapsed across Cue-Element, revealed a significant main effect of Session, $F(1,102) = 226.19$, $p < .01$, $\eta_p^2 = .69$, in addition to a significant interaction between Session and Loop, $F(1,102) = 27.204$, $p < .01$, $\eta_p^2 = .21$, with an overall greater decrease in accuracy across the elements in the open- relative to closed-loop condition between T1 and T2. No other significant main effect or interaction was observed, $F$s < 1.7, $p$s > .19.

Similarly, a 2x2x3 (Session x Loop x Cue-Element) ANOVA, collapsed across Retrieval-Element, revealed a significant main effect of Session, $F(1,102) = 226.18$, $p < .001$, $\eta_p^2 = .69$, in addition to a significant interaction between Session and Loop, $F(1,102) = 27.198$, $p < .001$, $\eta_p^2= .21$. We also saw a significant effect of Element, $F(1.86,190.64) = 9.92$, $p < .001$, $\eta_p^2 = .09$, in addition to a significant three-way interaction between Session x Element x Loop, $F(2,204) = 3.87$, $p = .02$, $\eta_p^2 = .04$. This interaction was characterised by a significant interaction between Element and Loop at T2, $F(2,204) = 5.48$, $p = .01$, $\eta_p^2 = .05$, with significantly greater retrieval accuracy for locations than people, $t(51) = 3.17$, $p = .01$, $d = .28$, and objects $t(51) = 4.14$, $p < .01$, $d = .35$, in the open-loop condition. No such effect was observed in the closed-loop condition when corrected for multiple comparisons.

The difference in retrieval accuracy across the elements suggests an underlying difference in memory across the elements. However, the difference in retrieval accuracy for the open-loop condition at T2, but not for closed-loops, suggests that when dependency is seen (as for the closed-loop condition), accuracy across the elements becomes more symmetrical.

**Retrieval accuracy for tested vs not-tested closed- and open-loops**

Table S3 provides mean proportion correct (and standard deviations) for retrieval accuracy at T2 dependent on whether the pairwise association had been previously tested at T1 or not.

**Table S3** Mean proportion correct (and standard deviations) at T2 for cue-target associations tested (*Tested*) and not tested (*Not tested*) previously at T1. For Experiment 1, 2, and 4. n/a = not applicable.

| | **Condition** | **Loop** | **Tested** | **Not tested** |
|---|---|---|---|---|
| **Experiment 1** | Sleep | Open | .77 (.16) | .61 (.16) |
| | - | Closed | .78 (.24) | .69 (.27) |
| | Awake | Open | .70 (.15) | .51 (.14) |
| | - | Closed | .70 (.22) | .54 (.23) |
| **Experiment 2** | n/a | Open | .60 (.17) | .41 (.14) |
| | n/a | Closed | .68 (.22) | .46 (.20) |
| **Experiment 4** | n/a | Open | .58 (.17) | .37 (.14) |
| | n/a | Closed | .70 (.21) | .50 (.19) |

A 2x2x2 (Tested x Loop x Sleep) ANOVA revealed a main effect of Tested, $F(1,100) = 408.87$, $p < .001$, $\eta_p^2 = .80$, with greater accuracy for associations previously tested at T1. A Tested x Sleep interaction was also seen, $F(1,100) = 15.16$, $p < .001$, $\eta_p^2 = .13$, with a greater sleep effect for associations not previously tested (i.e., prior testing decreased the effect of sleep). A Tested x Loop interaction was also seen, $F(1,100) = 13.69$, $p < .001$, $\eta_p^2 = .12$, with a greater testing effect (Previously tested > Not previously tested) for open- relative to closed-loops. A main effect of Sleep was also seen, $F(1,100) = 6.54$, $p = .01$, $\eta_p^2 = .06$, consistent with the Session x Sleep interaction seen in the main analysis above. No further significant effects or interactions were seen, *Fs* < 2.58,

*ps* >.11. Prior testing therefore increased performance at T2 relative to associations not previously tested at T1, and this effect was modulated by Sleep and Loop.

**Retrieval dependency for tested vs not-tested closed- and open-loops**

Mean dependency (and standard deviations) for the data, independent model and dependent model for open- and closed-loops, collapsed across sleep, for associations previously tested and not tested are presented in Table S4 A 2x2x2 (Tested x Loop x Sleep) ANOVA similarly revealed a significant main effect of loop, $F(1,100) = 44.29$, $p < .001$, $\eta_p^2 = .31$. No other significant effects or interactions were seen, $Fs < 1.89$, $ps > .17$.

**Table S4** Mean proportion of joint retrieval (and standard deviations) for the data and independent model at T2 for closed- and open-loops cue-target associations tested (*Tested*) and not tested (*Not tested*) previously at T1 for Experiment 1, 2, and 4. For Experiment 1, the proportion of joint retrieval is collapsed across the sleep and awake condition.

| | | **Tested** | | **Not tested** | |
|---|---|---|---|---|---|
| | **Loop** | **Data** | **Independent** | **Data** | **Independent** |
| **Experiment 1** | Open | .62 (.16) | .65 (.14) | .52 (.10) | .55 (.06) |
| | Closed | .76 (.16) | .72 (.18) | .69 (.15) | .66 (.17) |
| **Experiment 2** | Open | .51 (.07) | .53 (.06) | .56 (.08) | .55 (.08) |
| | Closed | .72 (.12) | .65 (.14) | .62 (.08) | .58 (.09) |
| **Experiment 4** | Open | .54 (.17) | .56 (.09) | .56 (.10) | .56 (.08) |
| | Closed | .71 (.14) | .66 (.14) | .60 (.09) | .57 (.07) |

**Dependency across element- and analysis-type**

We have previously shown that the proportion of joint retrieval in the observed data does not vary significantly across element-type (i.e., people, locations, and objects), and analysis type ($A_BA_C$, where the element A refers to the common cue element-type; and $B_AC_A$, where the element A refers to common retrieved element-type) (Horner & Burgess, 2014). In order to assess for differences in dependency that might be due to variations in the memorability of element-type across triplets, we repeated the main analysis reported for Experiment 1 across individual element-types for the two separate analysis types (i.e., $A_BA_C$ and $B_AB_C$).

**Table S5** Mean proportion of joint retrieval (and standard deviations) for data and independent model across element-type for test session T2 for experiment 1 across analysis-types $A_BA_C$ and $B_AC_A$. Proportion of joint retrieval is collapsed across the sleep and awake conditions. For T2, only trials where participants retrieved cue-target associations not previously tested at T1 are included. Element-types (i.e., people, locations, and objects) refer to the type of common cue or retrieval element in the dependency analysis-types $A_BA_C$ and $B_AC_A$ (i.e., element A in $A_BA_C$ and $B_AC_A$), respectively.

| | | | **Session** | | | |
|---|---|---|---|---|---|---|
| | | | **T1** | | **T2** | |
| **Analysis** | **Loop** | **Element** | **Data** | **Independent** | **Data** | **Independent** |
| $A_BA_C$ | Open | People | .67 (.20) | .65 (.15) | .55 (.17) | .55 (.10) |
| - | - | Locations | .63 (.22) | .65 (.16) | .57 (.20) | .58 (.12) |
| - | - | Objects | .59 (.17) | .61 (.14) | .52 (.16) | .53 (.09) |
| - | Closed | People | .72 (.17) | .68 (.18) | .69 (.16) | .66 (.16) |
| - | - | Locations | .73 (.18) | .69 (.19) | .70 (.16) | .65 (.17) |
| - | - | Objects | .72 (.16) | .67 (.17) | .70 (.19) | .66 (.18) |
| $B_AC_A$ | Open | People | .63 (.18) | .64 (.14) | .52 (.17) | .54 (.10) |
| - | - | Locations | .64 (.20) | .66 (.16) | .48 (.16) | .54 (.12) |
| - | - | Objects | .58 (.19) | .61 (.15) | .50 (.15) | .54 (.08) |
| - | Closed | People | .71 (.19) | .68 (.18) | .71 (.17) | .66 (.17) |
| - | - | Locations | .68 (.20) | .67 (.18) | .68 (.17) | .65 (.17) |
| - | - | Objects | .71 (.19) | .68 (.18) | .68 (.16) | .65 (.16) |

Mean proportion of joint retrieval (and standard deviations) in the data and independent model across test sessions T1 and T2 for analysis-type $A_BA_C$ and analysis-type $B_AC_A$ for Experiment 1 are reported in Table S5. A 2x2x3 (Session x Loop x Cue-type) ANOVA on dependency, collapsed across retrieval-type (i.e., the different elements-types in the $B_AB_C$ analysis) and the sleep and awake condition, replicated the main effect of Loop, $F(1,102) = 20.7$, $p < .001$, $\eta_p^2 = .17$. No other main effects or interactions were observed, *F*s < 1.87, *p*s > .15. Cue-type refers to the different element-type (i.e., people, locations, and objects) in the $A_BA_C$ analysis.

Similarly, 2x2x3 (Session x Loop x Retrieval-type) ANOVA on dependency, collapsed across cue-type (i.e., the different element-types in the $A_BA_C$ analysis) and the sleep and awake condition, replicated the main effect of

Loop, $F(1,102) = 36.86$, $p < .001$, $\eta_p^2 = .27$. The ANOVA failed to reveal any other significant main effects or interactions, $F$s < 2.17, $p$s > .11, although we note a borderline interaction between Session and Loop, $F(1,102) = 3.42$, $p < .07$, $\eta_p^2 = .32$. No other main effects or interactions were observed, $F$s < 2.17, $p$s > .11. Retrieval-type refers to the different element-type (i.e., people, locations, and objects) in the $B_AC_A$ analysis.

**Dependency for closed-loops including only two pairwise associations**

We have previously shown that closed-loops show significantly greater dependency than open-loops, even when the number of learnt elements or associations between open- and closed-loops is equated (Horner & Burgess, 2014). For consistency, we repeated the main dependency analysis for closed-loops reported above, but including only two out of the three possible pairwise associations. As such, the number of retrieval trials included in the analysis is equivalent to those included in the dependency analysis for open-loops reported in the main analysis. Consistent with previous published work (Horner & Burgess, 2014), despite including only two out of the three learned associations, closed-loops show significant dependency at both T1, $t(51) = 4.78$, $p < .001$, $d = .16$ and T2, $t(51) = 4.94$, $p < .001$, $d = .21$. Similarly, a 2x2x2 (Session x Loop x Sleep) ANOVA replicated the significant effect of loop, $F(1,100) = 31.29$, $p < .001$, $\eta_p^2 = .24$ reported in the main analysis. No other significant main effects or interactions were observed, $Fs$ < 2.26, $ps$ > .13. A 2x2x2 (Tested x Loop x Sleep) ANOVA also replicated the main effect of Loop, $F(1,100) = 36.33$, $p < .001$, $\eta_p^2 = .27$, in addition to a significant interaction between Tested and Sleep, $F(1,100) = 4.72$, $p = .03$, $\eta_p^2 = .05$, with a greater difference between dependency for previously tested vs not tested associations in the awake condition relative to participants in the sleep condition.

**Experiment 2**

**Retrieval accuracy for tested vs not-tested closed- and open-loops**

A 2x2 (Tested x Loop) ANOVA on retrieval accuracy at T2 revealed a main effect of Tested, $F(1,50) = 187.48$, $p < .001$, $\eta_p^2 = .79$, with greater accuracy for previously tested than not previously tested associations. No other significant main effects or interactions were observed, $Fs$ < 1.87, $ps$ > .17.

**Retrieval dependency for tested vs not-tested closed- and open-loops**

A 2x2 (Tested x Loop) ANOVA on dependency at T2 revealed a significant main effect of Loop, $F(1,50) = 78.37$, $p < .001$, $\eta_p^2 = .61$, in addition to a significant Tested x Loop interaction, $F(1,50) = 14.44$, $p < .001$, $\eta_p^2 = .22$, with a

greater difference in dependency between closed- and open-loops previously tested at T1, relative to not tested at T1.

**Experiment 4**

**Retrieval accuracy for tested vs not-tested closed- and open-loops**

A 2x2 (Tested x Loop) ANOVA on retrieval accuracy at T2 revealed a main effect of Tested, $F(1,25) = 142.12$, $p < .001$, $\eta_p^2 = .85$, with greater accuracy for previously tested, relative to closed- and open-loops not tested previously. The ANOVA also revealed a main effect of Loop, $F(1,25) = 50.03$, $p < .001$, $\eta_p^2 = .67$ with overall greater retrieval accuracy for closed-loops than open-loops.

**Retrieval dependency for tested vs not-tested closed- and open-loops**

A 2x2 (Tested x Loop) ANOVA on dependency revealed a main effect of Loop, $F(1,25) = 14.21$, $p < .001$, $\eta_p^2 = .36$. No other significant effects or interactions were observed, $Fs < 2.83$, $ps > .96$.
